# Supplementary material for: Effects of helminths and anthelmintic treatment on cardiometabolic diseases and risk factors: A systematic review
Source: PLoS Negl Trop Dis. 2023 Feb 24;17(2):e0011022. doi: 10.1371/journal.pntd.0011022 (PMC9956023; doi:10.1371/journal.pntd.0011022)
Supplement: S1 Table — (DOCX) [file pntd.0011022.s001.docx]

| **Cardiometabolic Disease and Risk Factors** | **Major Causes of Human Helminthiases** | **Study Types** | **Metabolic Syndrome** |
| --- | --- | --- | --- |
| Cardiomyopathies | *Ancylostoma duodenale, Necator americanus* | Animal and human | Abdominal obesity |
| Cerebrovascular disease | *Ascaris lumbricoides* | Case-control | Atherogenic dyslipidemia |
| Congenital heart disease | *Brugia malayi,*  *Wuchereria bancrofti* | Cohort | Glucose intolerance, insulin resistance |
| Coronary heart disease | *Clonorchis sinensis, Opisthorchis viverinni* | Cross-sectional | Raised blood pressure |
| Diabetes | *Dracunculus medinensis* | Randomized clinical trial |  |
| Heart failure | *Fasciola hepatica,* |  |  |
| High-density lipoprotein cholesterol | *Loa loa*, *Onchocerca volvulus* |  |  |
| High-sensitivity c-reactive protein | *Paragonimus* spp |  |  |
| Hypertension | *Schistosoma haematobium, Schistosoma japonicum, Schistosoma mansoni* |  |  |
| Peripheral vascular disease | *Strongyloides stercoralis* |  |  |
| Rheumatic heart disease | *Taenia solium, Fasciolopsis buski* |  |  |
| Systolic blood pressure | *Trichuris trichiura* |  |  |
| Total cholesterol |  |  |  |
