# Supplementary material for: Effects of helminths and anthelmintic treatment on cardiometabolic diseases and risk factors: A systematic review
Source: PLoS Negl Trop Dis. 2023 Feb 24;17(2):e0011022. doi: 10.1371/journal.pntd.0011022 (PMC9956023; doi:10.1371/journal.pntd.0011022)
Supplement: S2 Table — Abbreviations: IQR, interquartile range; STH, soil-transmitted helminths; HDL, high-density lipoprotein; TC, total cholesterol; LDL, low-density lipoprotein; trig, triglycerides; PCR, polymerase chain reaction; RCT, randomized controlled trial; PZQ, praziquantel; SEA, soluble egg antigen; CAD, coronary artery disease; PSI, previous schistosome infection; T2DM, type 2 diabetes; HFD, high-fat diet; BMI, body mass index. #study investigated other outcome measures that will be included in other tables. *denotes statistical significance, p<0.05. (DOCX) [file pntd.0011022.s002.docx]

| **Overview:**   - 27 human and 17 animal studies, 1 mixed study - Estimated median sample size = 167.5 [IQR 41-624.5] - Helminths represented: *S. mansoni* (14), mixed STH (5), *S. japonicum* (5), unspecified *Schistosoma* species (5), *F. hepatica* or unspecified *Fasciola* species (4), *O. viverinni or* unspecified *Opisthorchis* species *(4)*, *S. haematobium* (2), *S. stercoralis* (2), *A. lumbricoides* (1), *C. sinensis* (1), mixed filarial species *(W. bancrofti, B. malayi*) (1), and *O. volvulus* (1) - Only 6 of 45 studies reported on both baseline and follow-up serum lipids (before and after anthelmintic treatment) - Human studies:   - 22 cross-sectional, 2 randomized clinical trials, 1 autopsy, 1 prospective cohort, 1 mixed human/animal, and 1 combined cross-sectional and interventional study  - Median age: 44.2 [IQR 32-65.1] years  - Median percent of women: 47.1% [IQR 27.7-51.3]   - Animal studies:   - 13 mouse, 2 sheep, 1 rabbit, 1 hamster, 1 primate (including mixed study)  - Only 11 clearly reported on distribution of sex with 9 using all male and 2 using all female animals | | | | | | | | |
| --- | --- | --- | --- | --- | --- | --- | --- | --- |
| **Study, Year (reference #)** | **Study type (animal model, method of infection/diagnosis)** | **Country** | **Parasite Species** | **Outcome** | **Sample Size** | **Sex (% Female)** | **Age in Years (Mean or Median)** | **Effect of Parasitic Infection and Anthelmintic Treatment on Outcome** |
| **Studies examining serum lipids before and after anthelmintic treatment (n=6)** | | | | | | | | |
| **Human studies (n=3)** | | | | | | | | |
| Muthukumar, 2020 (14) | Human (stool microscopy, prospective cohort | Thailand | *O. viverrini* | Lipid profile^#^ | 400 | 60% | Unclear  (age≤50: n=219; age≥51: n=181) | Baseline  ↑ HDL*; no differences in TC, LDL, or trig  Follow-up  ↑ HDL*; ↑ LDL + ↓ TC and trig in infected individuals 6 months after PZQ |
| Sanya, 2020 (13) | Human (stool microscopy and PCR), cluster-RCT | Uganda | Mixed helminths  (*S. mansoni, S. stercoralis, T. trichiura*) | Lipid profile^#^ | 1898 | 46.5% vs. 47.1%  (intensive vs. standard anthelmintic treatment) | 32 vs. 31 years  (intensive vs. standard anthelmintic treatment) | Baseline  ↓ LDL* with *S. mansoni* or *Strongyloides* infections; ↓ TC* with *S. mansoni* and ↓ LDL* and trig* with moderate-heavy *S. mansoni* infection; no difference in HDL  Follow-up  ↑ mean LDL with intensive treatment with PZQ/albendazole after 4 years of follow-up (not significant, p=0.08) |
| Tahapary, 2017 (8) | Human (stool microscopy, stool PCR), cluster-RCT | Indonesia | Mixed helminths  (*A. lumbricoides, T.* *trichiura, S. stercoralis*) | Lipid profile^#^ | 1669 | 60% vs. 61.2% (albendazole treatment vs. placebo) | 42.5 vs. 42.5 years (albendazole treatment vs. placebo) | Baseline  No differences in TC, LDL, HDL, trig (albendazole vs. placebo)  Follow-up  No effect after 52 weeks of follow-up |
| **Animal studies (n=3)** | | | | | | | | |
| Kozat, 2010  (17) | Animal (Akkaraman sheep; stool microscopy) | Turkey | *F. hepatica* | Lipid profile^#^ | 25 | Not reported | 3-5 years | Baseline  ↓ TC*, trig*, LDL*, HDL*  Follow-up lipids ↑ but remained lower than controls on day 28 in infected sheep treated with triclabendazole/levamisole (day 0); no differences between infected and control groups at day 56, except infected, treated sheep still had lower HDL* |
| Luo, 2017  (15) | Animal (C57BL/6 and diabetes db mutation of the leptin receptor (Lepr ^db/db^) mice; cercariae and SEA) | China | *S. japonicum* | Lipid profile^#^ | 90 | 0%  (appears only male mice were used) | 6 weeks | Baseline  No differences in TC, LDL, HDL, or trig  Follow-up  ↑ LDL* and HDL* 9 weeks after PZQ vs. infected/control CMC mice and uninfected mice; no effect on TC or trig |
| Yuksek, 2013  (16) | Animal (Akkaraman sheep; stool microscopy) | Turkey | *Fasciola* species | Lipid profile^#^ | 30 | Not reported | 1-3 years | Baseline  ↓ LDL*; no difference in TC, HDL, trig  Follow-up  ↑ TC*, LDL* HDL*; ↓ trig* 28 days after infected sheep were treated with triclabendazole/levamisole |
| **Studies examining serum lipids only cross-sectionally (n=39)** | | | | | | | | |
| **Human studies (n=24)** | | | | | | | | |
| Afshan, 2020 (20) | Human (*Fasciola* IgG Enzyme Immunoassay), cross-sectional | Pakistan | *Fasciola* species | Serum TC^#^ | 100 | Unclear total but 6.6% infection prevalence in women | Unclear (reports age ranges from 10 to 69 years) | ↑ TC* |
| Aravindhan, 2012  (22) | Human (serum filarial antigen and IgG + IgG4 antibody), cross-sectional | India | Mixed filarial species (*W. bancrofti* and *Brugia malayi*) | Lipid profile^#^ | 453 | 34.5% | 51.5 vs. 51.7 years  (CAD+ group vs. CAD- group) | No difference in TC, LDL, HDL, or trig |
| Buck, 1971  (23) | Human (urine microscopy), cross-sectional | Republic of Chad | *O. volvulus* | Serum TC^#^ | 153 | 48% | Unclear (reports age ranges from 0 to > 40 years) | No difference in TC—166.7 vs. 152.2 mg/mL in those with and without microfilaruria, respectively (borderline, p=0.07) |
| Changbumrung, 1988 (24) | Human (stool microscopy), cross-sectional | Thailand | *O. viverrini* | Lipid profile^#^ | 42 | Not reported | Unclear (reports age ranges from 14-69 years) | ↓ TC* and no difference in trig vs. controls; serum LDL and HDL comparisons not clearly made |
| Chen, 2013 (25) | Human (study-defined PSI criteria), cross-sectional | China | *Schistosoma* species | Lipid profile^#^ | 3913 | 47.1 vs. 61.4% (with PSI vs. without PSI)* | 70.5 vs. 67.6 years (with PSI vs. without PSI)* | ↓ TC*, LDL*, and trig*; ↑ HDL* |
| da Silva, 2018 (54) | Human (stool microscopy), cross-sectional | Brazil | *S. mansoni* | Lipid profile | 1078 | Unclear total but 28.2% infection prevalence in females | Unclear (reports age ranges from 0 to > 71 years) | ↓ TC*, trig*, and HDL* + no difference in LDL |
| Dessie, 2020 (26) | Human (stool microscopy), cross-sectional | Ethiopia | *S. mansoni* | Serum TC^#^ | 220 | 50% | 30.9 vs. 31.1 years (infected vs. control) | ↓ TC* |
| El-Shazly, 2008 (29) | Human (stool microscopy), cross-sectional | Egypt | *Fasciola* species | Lipid profile | 69 | Not reported | Not reported | ↓ TC*, LDL*, HDL* and ↑ trig* with higher intensities of infection |
| Gillett, 1978  (31) | Human (stool microscopy), cross-sectional | Brazil | *S. mansoni* | Plasma TC | 93 | Not reported | Not reported | ↓ TC* in compensated and decompensated hepatosplenic schistosomiasis vs. controls |
| Hays, 2015 (32) | Human (parasite IgG antibody), cross-sectional | Australia | *S. stercoralis* | Serum TC, HDL, trig^#^ | 259 | 59.1% | 43.4 | ↑ median trig* in T2DM participants (higher +IgG in these people); no difference in TC or HDL; LDL not studied |
| Ines, 2017 (34) | Human (stool microscopy), cross-sectional | Brazil | *S. stercoralis* | Lipid profile | 276 | Not reported | 43.9 vs. 47.0 years (alcoholic vs. non-alcoholic individuals) | ↓ trig* and LDL* in alcoholic individuals; ↑ TC* and LDL* but no effect on trig in non-alcoholic individuals; ↑ HDL* in both groups |
| Li, 2016  (6) | Human (study defined criteria for hepatosplenic disease + stool microscopy used to exclude active infection; cross-sectional | China | *S. japonicum* | Lipid profile^#^ | 82 | 57% | 73.7 vs. 72.6 (infected vs. controls) | ↑ TC* and LDL* in chronic hepatic schistosomiasis *japonicum* patients with insulin resistance (IR) with diabetes (DM) than controls; ↓ HDL* in those with chronic HSJ and IR without DM than controls; no difference or effect in triglycerides |
| Magen, 2013  (38) | Human cadaver/autopsy (liver examination for worms) | Russia | *Opisthorchis* species | Serum TC^#^ | 319 | 12.2% | Unclear (reports age ranges from 20 to > 60 years) | ↓ TC* |
| Mohamed, 2017  (56) | Human (stool microscopy to rule out active infection; study-defined PSI criteria), cross-sectional | Egypt | *Schistosoma* species | HDL and trig^#^ | 574 | 27.7% | 56.7 vs, 57.9 (infected vs. uninfected) | ↓ HDL* and trig* |
| Onuegbu, 2011 (39) | Human (urine microscopy), cross-sectional | Nigeria | *S. haematobium* | Lipid profile | 200 | 50% | 11.0 vs. 10.5 (infected vs. uninfected) | ↓ TC*, trig*, HDL*, and LDL*; ↓ TC* in males vs. females with infection |
| Sanya, 2020  (7) | Human (stool microscopy, stool PCR), cross-sectional | Uganda | Mixed helminths (*S. mansoni, T. trichiura, A. lumbricoides, S. stercoralis*) | Lipid profile^#^ | 2828 | 49% vs. 65% (rural vs. urban survey)* | 31.5 vs. 29.7 years (rural vs. urban survey)* | No differences in lipids between rural and urban residents (current helminth infection did not explain for small differences) |
| Shen, 2014  (43) | Human (study-defined PSI criteria), cross-sectional | China | *Schistosoma* species | Lipid profile^#^ | 1942 | With PSI: 11.6%  Without PSI: 20.1% | 65.7 vs. 64.9 years (men with PSI vs. without PSI)  64.4 vs. 65.4 years (women with PSI vs. without PSI) | ↓ trig* and ↑ HDL* among those with PSI; no difference in TC or LDL |
| Shen, 2015  (42) | Human (study-defined PSI criteria + stool microscopy used to exclude active infection), cross-sectional | China | *Schistosoma* species | Lipid profile^#^ | 1597 | None (only men enrolled) | 65.7 vs. 64.9 years (with PSI vs. without PSI) | ↓ trig* and ↑ HDL* among those with PSI; no difference in TC or LDL |
| Tahapary, 2018  (45) | Human (stool PCR), cross-sectional and interventional study (exposure to a HFD) | Indonesia | Mixed helminths (*N. americanus, A. duodenale, A. lumbricoides, T. trichiura, S. stercoralis*) | LDL^#^ | 154 | 0% (only men enrolled) | 44.5 vs. 39.3 (rural vs. urban) | ↓ LDL*  ↑ LDL (not significant) among infected individuals vs. uninfected individuals after short-term HFD challenge |
| Wiria, 2013  (47) | Human (stool microscopy with stool PCR), cross-sectional | Indonesia | Mixed helminths (*T. trichiura, A. lumbricoides, N. americanus, A. duodenale, S. stercoralis*) | Lipid profile^#^ | 675 | 62.3% vs. 65.9% (infected vs. uninfected) | 45.0 vs. 44.8 years (infected vs. uninfected) | ↓ TC* and LDL* (attenuated after BMI adjustment); no difference in HDL or trig |
| Wolde, 2019  (48) | Human (stool microscopy, cross-sectional | Ethiopia | *S. mansoni* | Lipid profile^#^ | 181 | *S. mansoni* positive (endemic): 41.5%  *S. mansoni* negative (endemic): 49.4%  *S. mansoni* negative (non-endemic): 29.5% | *S. mansoni* positive (endemic): 44.2  *S. mansoni* negative (endemic): 39.9  *S. mansoni* negative (non-endemic): 28.1 | ↓ TC, LDL*, HDL*, and overall dyslipidemia in infected vs. uninfected people in both regions; ↓ trig* in infected individuals in endemic vs. uninfected individuals in nonendemic region, but no difference in trig levels within the same region |
| Zaman, 2018  (21) | Human (parasite IgG antibody), cross-sectional | Pakistan | *A. lumbricoides* | Serum TC^#^ | 356 | 47% | 22.3 years | ↑ TC* |
| Zinsou, 2020  (52) | Human (urine microscopy), cross-sectional | Gabon | *S. haematobium* | Serum lipid profile^#^ | 71 | 51.3% vs. 56.2% (infected vs. uninfected) | 34.5 vs. 35.7 years (infected vs. uninfected) | ↓ HDL* and trig* and no differences in TC or LDL; however, effects were only seen in overweight/obese infected individuals when stratified by BMI |
| Zou, 2021  (53) | Human (study-defined PSI criteria), cross-sectional | China | *Schistosoma* species | Serum lipid profile^#^ | 2867 | 20.7% vs. 20.3% (PSI vs. without PSI) | 68.5 vs. 68.0 years (PSI vs. without PSI) | ↓ LDL* and trig* and ↑ HDL*; no difference in TC (p=0.053) |
| **Animal studies (n=14)** | | | | | | | | |
| De Oliveira, 1977  (55) | Animal (Swiss mice; cercariae) | Brazil | *S. mansoni* | Total cholesterol | Unclear, possibly 33 | None (only male mice used) | 4-5 months | ↓ TC* in infected mice vs. uninfected mice |
| Doenhoff, 2002  (27) | Animal (ApoE-deficient and random-bred TO mice; cercariae) | United Kingdom | *S. mansoni* | Lipid profile ^#^ | Unclear, possibly 71 | Unclear, possibly 52.1% | Not reported | ↓ TC*, LDL*, and HDL* in infected mice fed HFD; no difference in trig |
| Filomeno, 2020  (30) | Animal (C57BL/6 mice; cercariae) | Brazil | *S. mansoni* | Lipid profile^#^ | 33 | None (only male mice used) | Not reported | ↓ TC*, LDL*, and trig* + ↑ HDL* in infected mice fed HFD vs. uninfected mice fed HFD |
| Hong, 1994  (33) | Animal (New Zealand rabbits; metacercariae) | Republic of Korea | *C. sinensis* | Serum TC | 9 | None (only male rabbits used) | 2-4 month-old | ↑ TC* during first week, then ↓* to below baseline level during weeks 2-8 (no clear control group) |
| La Flamme, 2007  (35) | Animal (ApoE-deficient C57BL/6 mice; parasite egg exposure) | New Zealand | *S. mansoni* | Lipid profile^#^ | Unclear | Not reported | 6-10 weeks for lipid studies | ↓ TC* + LDL* in infected mice fed HFD vs. uninfected mice but no difference in trig or HDL |
| Laothong, 2013  (36) | Animal (Syrian golden hamsters; metacercariae) | Thailand | *O. viverrini* | Lipid profile | 60 | None (only male hamsters used) | 4-6 weeks | ↓ HDL* and HDL/cholesterol ratio* throughout acute/chronic phases of infection; ↑ TC* and LDL* in acute phase, ↑ trig* in chronic phase |
| Lira, 2019  (37) | Animal (BALB/c mice; cercariae) | Brazil | *S. mansoni* | Lipid profile^#^ | 40 | 100% (only female mice used) | Not reported | ↓ HDL* and trig* in infected animals fed normal and HFD (only in normal diet for trig finding); no significant difference in TC; LDL not studied |
| Owen, 1978  (40) | Animal (Swiss mice; cercariae) | Unclear, possibly Brazil | *S. mansoni* | Plasma TC, trig | 16 | None (only male mice used) | Not reported | ↓ TC*;  no difference in trig |
| Ramos, 2004  (41) | Animal (Callithrix jacchus; cercariae) | Brazil | *S. mansoni* | Plasma TC and trig | 11 | None (only male primates used) | Not reported | ↓TC* and trig* with both first infection and re-infection |
| Stanley, 2009  (44) | Animal (ApoE-deficient and random bred TO-strain mice; cercariae, SEA, or whole eggs | United Kingdom | *S. mansoni* | Serum TC | Unclear, possibly 106 | Not reported | Not reported | ↓ TC* in mice exposed to whole eggs or SEA, but not to cercariae |
| Toulah, 2018  (46) | Animal (Swiss albino mice; SEA or cercariae) | Egypt | *S. mansoni* | Lipid profile^#^ | 40 | 0% (only male mice used) | 6-8 weeks | ↓ TC*, trig*, HDL*, and LDL* in mice on HFD exposed to either SEA or cercariae |
| Wolfs, 2014  (49) | Animal (C57BL/6 wild-type and LDL R^-/-^ mice; SEA) | The Netherlands | *S. mansoni* | Serum TC and trig^#^ | Unclear, possibly 20-40 | Not reported | Not reported | ↓ TC after 5 and 10 weeks of HFD*; no difference in triglycerides |
| Xue, 2014  50) | Animal (ICR mice; cercariae | China | *S. japonicum* | Lipid profile | 24 | Not reported | Not reported | ↓ serum TC*, LDL*, HDL*, and trig* in infected mice on a high-fat or normal diet vs. controls |
| Yang, 2021  (51) | Animal (ApoE-deficient C57BL/6 mice; parasite recombinant enzyme, rSj-Cys) | China | *S. japonicum* | Lipid profile^#^ | 24 | 0% (only male mice used) | 7-8 weeks old | ↓ TC*, LDL*, and trig* + ↑ HDL* associated with exposure to rSj-Cys in mice fed HFD vs. unexposed mice fed HFD |
| **Mixed studies (n=1)** | | | | | | | | |
| Duan, 2018  (28) | Mixed animal (C57BL/6 and ob/ob mice; cercariae) and human (chronic schistosomiasis; unclear method of diagnosis) | China | *S. japonicum* | Lipid profile^#^ | 2183 (human) | Humans: 24.3% vs. 25.1% (infected vs. controls) | 51.7 vs. 49.5 years (human infected patients vs. controls) | Humans: ↓ TC*, LDL*, and triglycerides* and ↑ HDL* |
