# Supplementary material for: Effects of helminths and anthelmintic treatment on cardiometabolic diseases and risk factors: A systematic review
Source: PLoS Negl Trop Dis. 2023 Feb 24;17(2):e0011022. doi: 10.1371/journal.pntd.0011022 (PMC9956023; doi:10.1371/journal.pntd.0011022)
Supplement: S3 Table — Abbreviations: IQR, interquartile range; STH, soil-transmitted helminths; PCR, polymerase chain reaction; BMI, body mass index; T2DM, type 2 diabetes; WC, waist circumference; PZQ, praziquantel; IR, insulin resistance; RCT, randomized controlled trial; HOMA-IR, homeostatic model assessment for insulin resistance; SEA, soluble egg antigen; PSI, previous schistosome infection; FBG, fasting blood glucose;; ART, antiretroviral therapy; HFD, high-fat diet; WHR, waist-hip ratio. #study investigated other outcome measures that will be included in other tables. *denotes statistical significance, p<0.05. (DOCX) [file pntd.0011022.s003.docx]

| **Overview:**   - 24 human and 13 animal studies, 1 mixed study - Estimated median sample size = 213.5 [IQR 71-1597] - Helminths represented: *S. mansoni* (9), *S. japonicum* (5), unspecified *Schistosoma* species (5), mixed STH (7), *S. stercoralis* (5), *F. hepatica* or *Fasciola* species (4), *A. lumbricoides* (1), *O. viverrini* (1) and *S. haematobium* (1) - Only 10 of 34 studies reported on both baseline and follow-up metabolic syndrome or related parameters (before and after anthelmintic treatment) - Human studies:   - 17 cross-sectional, 4 prospective cohorts, 2 randomized clinical trials, 1 combined cross-sectional and interventional study, and 1 mixed human/animal study  - Median age: 44.2 years [IQR 36-56.7]  - Median percent of women: 49.5% [IQR 41.5-57]   - Animal studies:   - 11 mouse, 3 sheep (including mixed study)  - Only 8 clearly reported on distribution of sex with 5 using all male and 3 using all female animals | | | | | | | | |
| --- | --- | --- | --- | --- | --- | --- | --- | --- |
| **Study, Year (reference #)** | **Study type (animal model, method of infection/diagnosis)** | **Country** | **Parasite Species** | **Outcome** | **Sample Size** | **Sex (% Female)** | **Age in Years (Mean or Median)** | **Effect of Parasite and Anthelmintic Treatment on Outcome** |
| **Studies examining metabolic syndrome before and after anthelmintic treatment (n=10)** | | | | | | | | |
| **Human studies (n=6)** | | | | | | | | |
| Hays, 2017  (57) | Human (serum parasite IgG antibody, stool PCR), prospective cohort | Australia | *S. stercoralis* | BMI, random blood glucose^#^ | 207 | Not reported | Unclear (reports age ranges from < 30 to > 50 years) | Baseline  No difference in BMI or random blood glucose between treated (infected) vs. not treated (uninfected) among those with or without T2DM  Follow-up  No effect on BMI or random blood glucose 3 years after infected individuals were treated with ivermectin |
| Muthukumar, 2020  (14) | Human (stool microscopy, prospective cohort | Thailand | *O. viverrini* | BMI, WC^#^ | 400 | 60% | Unclear  (age≤50: n=219; age≥51: n=181) | Baseline  No difference in BMI, WC  Follow-up  No effect on BMI, WC in infected individuals 6 months after PZQ |
| Rajamanickam, 2019  (58) | Human (parasite IgG antibody, stool microscopy); prospective cohort | India | *S. stercoralis* | IR and serum glucose^#^ | 118 | 50% vs. 48.3% (infected vs. uninfected) | Median of 46 vs. 45 years (infected vs. uninfected) | Baseline  No difference in random blood glucose, but ↓ insulin level* in infected group with T2DM vs. uninfected with T2DM  Follow-up  ↑ random blood glucose by 18%* and ↑ insulin level by 13%* in infected individuals compared to pretreatment levels (6 months after ivermectin/albendazole) |
| Rajamanickam, 2020  (59) | Human (parasite IgG antibody, stool microscopy); prospective cohort | India | *S. stercoralis* | Plasma insulin level, BMI, random blood glucose | 115 | 48.3% vs. 50.9% (infected vs. uninfected) | Median of 36 vs. 39 years (infected vs. uninfected) | Baseline  ↓ insulin level*; no difference in BMI or random blood glucose between infected vs. uninfected non-diabetic, obese individuals  Follow-up  ↑ insulin level by 9%* in infected individuals compared to pretreatment levels (6 months after ivermectin/albendazole) |
| Sanya, 2020  (13) | Human (stool microscopy and PCR), cluster-RCT | Uganda | Mixed helminths (*S. mansoni, S. stercoralis, T. trichiura*) | HOMA-IR, fasting blood glucose^#^ | 1898 | 46.5% vs. 47.1% (intensive vs. standard anthelmintic treatment) | 32 vs. 31 years (intensive vs. standard anthelmintic treatment) | Baseline  No difference in HOMA-IR or fasting blood glucose  Follow-up  No effect on HOMA-IR or fasting blood glucose in either treatment groups (PZQ/albendazole) |
| Tahapary, 2017  (8) | Human (stool microscopy, stool PCR), cluster-RCT | Indonesia | Mixed helminths  (*A. lumbricoides, T.* *trichiura, S. stercoralis*) | IR and serum glucose^#^ | 1669 | 60% vs. 61.2% (albendazole treatment vs. placebo) | 42.5 vs. 42.5 years (albendazole treatment vs. placebo) | Baseline  No differences in fasting insulin or glucose levels or in HOMA-IR  Follow-up  ↑ HOMA-IR*in albendazole group after 52 weeks of follow-up; effect was greater in comparison to subjects without helminth infections at baseline (p = .01 for the interaction between helminth infection status at baseline and post-treatment). |
| **Animal studies (n=4)** | | | | | | | | |
| Kozat, 2010  (17) | Animal (Akkaraman sheep; stool microscopy) | Turkey | *F. hepatica* | Serum glucose^#^ | 25 | Not reported | 3-5 years | Baseline  ↓ glucose*  Follow-up  ↑ glucose* in infected group after treatment with triclabendazole/levamisole but remained lower than controls on day 28 + 56 |
| Luo, 2017  (15) | Animal (C57BL/6 and diabetes db mutation of the leptin receptor (Lepr ^db/db^) mice; cercariae and SEA) | China | *S. japonicum* | IR, body weight^#^ | 90 | 0%  (appears only male mice were used) | 6 weeks | Baseline  ↓ body weight* and IR* in infected mice; mice exposed to SEA also had ↓ body weight* and IR* compared to normal mice  Follow-up  ↓ body weight* and IR* vs. control mice but compared to infected mice that were not treated, mice who were treated with PZQ had ↑ weight* and IR* at 9 weeks |
| Shaheen, 1989  (60) | Animal (cercariae; Swiss albino mice) | Egypt | *S. mansoni* | Blood glucose | Not reported | 0% (only male mice used) | Not reported | Baseline  ↓ blood glucose*  Follow-up  ↑ blood glucose* in infected animals 7 and 14 days after PZQ |
| Yuksek, 2013  (16) | Animal (Akkaraman sheep; stool microscopy) | Turkey | *Fasciola* species | Serum glucose^#^ | 30 | Not reported | 1-3 years | Baseline  ↓ glucose*  Follow-up  ↑ glucose* 28 days after infected sheep were treated with triclabendazole/levamisole |
| **Studies examining metabolic syndrome only cross-sectionally (n=28)** | | | | | | | | |
| **Human studies (n=18)** | | | | | | | | |
| Afshan, 2020  (20) | Human (Fasciola IgG Enzyme Immunoassay), cross-sectional | Pakistan | *Fasciola* species | Serum glucose^#^ | 100 | Unclear total but 6.6% infection prevalence in females | Unclear (reports age ranges from 10 to 69 years) | No difference in serum glucose |
| Chen, 2013  (25) | Human (study-defined PSI criteria), cross-sectional | China | *Schistosoma* species | Metabolic syndrome^#^ | 3913 | 47.1 vs. 61.4% (with PSI vs. without PSI)* | 70.5 vs. 67.6 years (with PSI vs. without PSI)* | ↓ prevalence of metabolic syndrome* (↓ HOMA-IR*, ↓FBG*; ↓ BMI* and WC*) |
| Dessie, 2020  (26) | Human (stool microscopy), cross-sectional | Ethiopia | *S. mansoni* | Serum glucose^#^ | 220 | 50% | 30.9 vs. 31.1 years (infected vs. control) | No difference in glucose |
| Hays, 2015  (32) | Human (serum parasite IgG antibody), cross-sectional | Australia | *S. stercoralis* | BMI^#^ | 259 | 59.1% | 43.4 | No difference in BMI |
| Li, 2016  (6) | Human (study defined criteria for hepatosplenic disease + stool microscopy used to exclude active infection; cross-sectional | China | *S. japonicum* | BMI, IR^#^ | 82 | 57% | 73.7 vs. 72.6 (infected vs. controls) | ↑ prevalence of IR* in those with chronic hepatosplenic *S. japonicum* and normal liver function with portal systemic shunting  No difference in BMI |
| Mohamed, 2017  (56) | Human (stool microscopy to rule out active infection; study-defined PSI criteria), cross-sectional | Egypt | *Schistosoma* species | Metabolic syndrome^#^ | 574 | 27.7% | 56.7 vs, 57.9 (infected vs. uninfected) | ↓ BMI * and waist circumference*, and FBG in those with PSI; ↓ prevalence of metabolic syndrome* (32.7% vs. 42.3%) in those with PSI |
| PrayGod, 2022  (69) | Human (stool and urine microscopy), cross-sectional | Tanzania | Mixed helminths (*S. mansoni + haematobium, A. lumbricoides, S. stercoralis, T. trichiura*) | Insulin level and HOMA-IR, beta-cell function, BMI, WC, fat mass | 1718 | 54.8% vs. 59.2% (approx. average of infected vs. uninfected) | 39.5 vs. 40.8 years (approx. average of infected vs. uninfected) | HIV-infected not on ART:  ↓ HOMA-IR* and fasting insulin* only in schistosome-infected people; ↓ fasting insulin* and HOMA-beta* in those with STH  HIV-uninfected:  ↓ BMI* with mixed helminth infection; ↓ WC* and fat mass* only in HIV-uninfected people with STH infection, not with schistosome infection; ↑ beta-cell function* ( ↑ overall insulin secretion index) in people with schistosome infection |
| Sanya, 2020  (7) | Human (stool microscopy, stool PCR), cross-sectional | Uganda | Mixed helminths (*S. mansoni, T. trichiura, A. lumbricoides, S. stercoralis*) | Metabolic syndrome^#^ | 2828 | 49% vs. 65% (rural vs. urban survey)* | 31.5 vs. 29.7 years (rural vs. urban survey)* | No differences in FBG or HOMA-IR  (↓ mean FBG* and HOMA-IR* in urban residents, but current helminth infection did not explain for these differences) |
| Shen, 2014  (43) | Human (study-defined PSI criteria), cross-sectional | China | *Schistosoma* species | Metabolic syndrome^#^ | 1942 | With PSI: 11.6%  Without PSI: 20.1% | 65.7 vs. 64.9 years (men with PSI vs. without PSI)  64.4 vs. 65.4 years (women with PSI vs. without PSI) | ↓ prevalence of hyperglycemia (29.96% vs 41.34%), obesity (8.37% vs 16.43%), and abdominal obesity (24.30% vs 41.78%) in men with PSI vs. controls (all, p<0.001)—not seen in women  ↓ BMI* in both men and women; ↓ WC* only in men and ↓ FBG* only in women |
| Shen, 2015  (42) | Human (study-defined PSI criteria + stool microscopy used to exclude active infection), cross-sectional | China | *Schistosoma* species | Metabolic syndrome^#^ | 1597 | 0% (only men enrolled) | 65.7 vs. 64.9 years (with PSI vs. without PSI) | ↓ prevalence of metabolic syndrome (p<0.001) and its components including central obesity (p<0.001) (BMI and WC) in men with PSI vs. controls |
| Tahapary, 2018  (45) | Human (stool PCR), cross-sectional and interventional study (exposure to a HFD) | Indonesia | Mixed helminths (*N. americanus, A. duodenale, A. lumbricoides, T. trichiura, S. stercoralis*) | IR, WC, and serum glucose^#^ | 154 | 0% (only men enrolled) | 44.5 vs. 39.3 (rural vs. urban) | No difference  (↓ IR* and ↓ WC * in infected or uninfected individuals living in rural area vs. uninfected urban individual) |
| Talukder, 2022  (68) | Human (parasite IgG antibody), cross-sectional | Australia | *S. stercoralis* | BMI^#^ | 536 | 55.1% vs. 54.2% (infected vs. uninfected) | 40.4 vs. 38.0 (infected vs. uninfected) | No difference in BMI |
| Wiria, 2013  (47) | Human (stool microscopy with stool PCR), cross-sectional | Indonesia | Mixed helminths (*T. trichiura, A. lumbricoides, N. americanus, A. duodenale, S. stercoralis*) | BMI and WHR^#^ | 675 | 62.3% vs. 65.9% (infected vs. uninfected) | 45.0 vs. 44.8 years (infected vs. uninfected) | ↓ BMI* (mean difference -0.66, 95%CI [-1.26,- 0.06], p=0.031), WHR* (-0.01, [-0.02, -0.00], p=0.011 |
| Wiria, 2015  (65) | Human (stool microscopy with stool PCR), cross-sectional | Indonesia | Mixed helminths (*T. trichiura, A. lumbricoides, N. americanus, A. duodenale, S. stercoralis)* | BMI and HOMA-IR^#^ | 646 | 62.0% vs. 66.2% (infected vs. uninfected) | 45.2 vs. 44.4 years (infected vs. uninfected) | ↓ BMI (23.2 vs 22.5 kg/m2)* and HOMA-IR (0.97 vs 0.81)* |
| Wolde, 2019  (48) | Human (stool microscopy), cross-sectional | Ethiopia | *S. mansoni* | BMI and FBG^#^ | 181 | *S. mansoni* positive (endemic): 41.5%  *S. mansoni* negative (endemic): 49.4%  *S. mansoni* negative (non-endemic): 29.5% | *S. mansoni* positive (endemic): 44.2  *S. mansoni* negative (endemic): 39.9  *S. mansoni* negative (non-endemic): 28.1 | ↓ BMI* and FBG * |
| Zaman, 2018  (21) | Human (parasite IgG antibody), cross-sectional | Pakistan | *A. lumbricoides* | Serum glucose^#^ | 356 | 47% | 22.3 years | No difference in glucose |
| Zinsou, 2020  (52) | Human (urine microscopy), cross-sectional | Gabon | *S. haematobium* | HOMA-IR and serum and glucose | 71 | 51.3% vs. 56.2%. (infected vs. uninfected) | 34.5 vs. 35.7 years (infected vs. uninfected) | No difference in HOMA-IR or glucose |
| Zou, 2021  (53) | Human (study-defined PSI criteria), cross-sectional | China | *Schistosoma* species | BMI^#^ | 2867 | 20.7% vs. 20.3% (PSI vs. without PSI) | 68.5 vs. 68.0 years (PSI vs. without PSI) | No difference in BMI |
| **Animal studies (n=9)** | | | | | | | | |
| Cortes-Selva, 2018  (61) | Animal (C57BL/6 and ApoE-deficient mice; cercariae) | United States | *S. mansoni* | Glucose tolerance^#^ | Unclear | Unclear, but possibly only male mice used | Unclear, possibly 6 weeks of age | ↑ (improved) glucose tolerance* among infected mice fed normal or high-fat chow |
| Filomeno, 2020  (30) | Animal (C57BL/6 mice; cercariae) | Brazil | *S. mansoni* | Glucose tolerance^#^ | 33 | 0% (only male mice used) | Not reported | ↑ (improved) glucose tolerance* |
| Hussaarts, 2015  (62) | Animal (SEA and cercariae; C57BL/6J mice) | The Netherlands | *S. mansoni* | IR and body weight | Unclear | 0% (only male mice used) | 8-10 weeks | ↓ body weight* and IR* |
| Lira, 2019 (37) | Animal (BALB/c mice; cercariae) | Brazil | *S. mansoni* | Serum glucose, body weight^#^ | 40 | 100% (only female mice used) | Not reported | ↓ serum glucose*; ↓ body weight* in the last two weeks before euthanasia |
| Phiri, 2007  (63) | Animal (Scottish Blackface and Suffolk cross sheep; metacercariae) | Unclear, possibly Scotland | *Fasciola* species | Serum glucose and weight gain | 28 | Not reported | Approximately 11-18 months | ↓ weight* between 6-8 and 11 weeks post infection (wpi) in infected sheep; ↓ serum glucose * from 6 wpi to the end of the experiment in *F.* *hepatica*-infected sheep and from 9-11 wpi in *F. gigantica*-infected sheep |
| Saule, 2005  (66) | Animal (C57BL/6 mice; cercariae) | France | *S. mansoni* | Glucose and insulin tolerance test | Unclear | 100% (only female mice used) | 7 weeks | ↓ glucose tolerance* and ↑ insulinemia* from day 7 to day 21 |
| Tang, 2019  (70) | Animal (C57BL/6 and Lepr^db/db^ mice; SEA) | China | *S. japonicum* | IR and serum glucose | 24 | Not reported | 6 weeks | ↓ IR* and glucose levels* in mice treated with SEA |
| Thabet, 2008  (67) | Animal (Swiss albino mice; cercariae) | Egypt | *S. mansoni* | IR and serum glucose | 55 | Not reported | Not reported | No difference in serum insulin or glucose between infected and uninfected diabetic mice; ↓ serum insulin level* and ↑ glucose* in infected diabetic mice vs. control |
| Yang, 2021  (51) | Animal (ApoE-deficient C57BL/6 mice; parasite recombinant enzyme, rSj-Cys) | China | *S. japonicum* | Body weight^#^ | 24 | 0% (only male mice used) | 7-8 weeks old | ↓ body weight* + kidney weight in mice fed HFD receiving rSj-Cys vs. mice fed HFD not receiving rSj-Cys |
| **Mixed studies (n=1)** | | | | | | | | |
| Duan, 2018  (28) | Mixed animal (C57BL/6 and ob/ob mice; cercariae) and human (chronic schistosomiasis; unclear method of diagnosis) | China | *S. japonicum* | Glucose tolerance, serum glucose, and BMI^#^ | 2183 (human); unclear sample size of mice | Humans: 24.3% vs. 25.1% (infected vs. controls)  Mice—100% (only female mice used) | 51.7 vs. 49.5 years (human infected patients vs. controls)  10-12 weeks (mice) | Humans: ↓ BMI* and serum glucose*  Mice: ↑ (improved) glucose tolerance* and ↓ body weight* |
