# Supplementary material for: Effects of helminths and anthelmintic treatment on cardiometabolic diseases and risk factors: A systematic review
Source: PLoS Negl Trop Dis. 2023 Feb 24;17(2):e0011022. doi: 10.1371/journal.pntd.0011022 (PMC9956023; doi:10.1371/journal.pntd.0011022)
Supplement: S4 Table — Abbreviations: IQR, interquartile range; STH, soil-transmitted helminths; PCR, polymerase chain reaction; T2DM, type 2 diabetes mellitus; HbA1c, hemoglobin A1c; PZQ, praziquantel; LF, lymphatic filariasis; T1DM, type 1 diabetes mellitus; PSI, previous schistosome infection; DM, diabetes mellitus; ART, antiretroviral therapy; STZ, streptozotocin; NOD, non-obese diabetic; SEA, soluble egg antigen; PBS, phosphate buffered saline; SWA, soluble extracts of worms. #study investigated other outcome measures that will be included in other tables. *denotes statistical significance, p<0.05. (DOCX) [file pntd.0011022.s004.docx]

| **Overview:**   - 19 human and 11 animal studies - Estimated median sample size = 279.5 [IQR 118-842] - Helminths represented: *S. stercoralis* (8), *S. mansoni* (5), mixed STH (4), *S. japonicum* (3), unspecified *Schistosoma* species (3), mixed filarial species (3), *F. hepatica* (2), *B.* malayi (1), and *O. viverrini* (1) - Only 5 of 25 studies reported on both baseline and follow-up diabetes or related parameters (before and after anthelmintic treatment) - Human studies:   - 11 cross-sectional, 5 prospective cohorts, and 3 case-control studies  - Median age: 46 years [IQR 39.5-55.8]  - Median percent of women: 55% [IQR 49.2-59.6]   - Animal studies:   - All mouse studies  - Only 9 clearly reported on distribution of sex with 8 using all female and 1 using all male mice | | | | | | | | |
| --- | --- | --- | --- | --- | --- | --- | --- | --- |
| **Study, Year (reference #)** | **Study type (animal model, method of infection/diagnosis)** | **Country** | **Parasite Species** | **Outcome** | **Sample Size** | **Sex (% Female)** | **Age in Years (Mean or Median)** | **Effect of Parasite and Anthelmintic Treatment on Outcome** |
| **Studies examining diabetes before and after anthelmintic treatment (n=5)** | | | | | | | | |
| **Human studies (n=5)** | | | | | | | | |
| Hays, 2017  (57) | Human (serum parasite IgG antibody, stool PCR), prospective cohort | Australia | *S. stercoralis* | T2DM^#^ | 207 | Not reported | Unclear (reports age ranges from < 30 to > 50 years) | Baseline  ↓ risk of T2DM* (as reported in previous study, Hays 2015)  Follow-up  ↑ development of T2DM (not significant) and glucose intolerance* in those without T2DM at baseline; ↑ glycemic control* in those with pre-existing T2DM (3 years after infected individuals were treated with ivermectin) |
| Htun, 2018  (71) | Human (stool and urine microscopy, point-of-care and rapid diagnostic tests), prospective cohort | South Africa | Mixed helminths (*A. lumbricoides, T. trichiura, S. mansoni, S. haematobium*) | HbA1c | 842 | Not reported | 9-14 years | Baseline  No difference in HbA1c (except in those with *H. pylori*)  Follow-up  No effect on HbA1c (6 months after albendazole) |
| Muthukumar, 2020  (14) | Human (stool microscopy, prospective cohort | Thailand | *O. viverrini* | HbA1c^#^ | 400 | 60% | Unclear  (age≤50: n=219; age≥51: n=181) | Baseline  ↓ HbA1c* (5.5.% vs. 6.0%)  Follow-up  ↑ HbA1c* (5.5%-->6.0%) in infected individuals (6 months after PZQ) |
| Rajamanickam, 2019  (58) | Human (parasite IgG antibody, stool microscopy); prospective cohort | India | *S. stercoralis* | HbA1c^#^ | 118 | 50% vs. 48.3% (infected vs. uninfected) | Median of 46 vs. 45 years (infected vs. uninfected) | Baseline  No difference in hemoglobin A1c in infected vs. uninfected individuals with T2DM  Follow-up  ↑ hemoglobin A1c by 25%* in infected individuals compared to pretreatment levels (6 months after ivermectin/albendazole) |
| Rajamanickam, 2020  (59) | Human (parasite IgG antibody, stool microscopy); prospective cohort | India | *S. stercoralis* | HbA1c^#^ | 115 | 48.3% vs. 50.9% (infected vs. uninfected) | Median of 36 vs. 39 years (infected vs. uninfected) | Baseline  No difference in HbA1c between infected vs. uninfected non-diabetic, obese individuals  Follow-up  No reported effect on HbA1c in infected individuals (6 months after ivermectin/albendazole) |
| **Studies examining diabetes only cross-sectionally (n=25)** | | | | | | | | |
| **Human studies (n=14)** | | | | | | | | |
| Almugadam, 2021  (85) | Human (stool and urine microscopy), case-control | Sudan | *Schistosoma* species | T2DM | 300 | 50% | Unclear (reports age ranges 21-40, 41-60, 61-80) | ↑ odds of testing positive for urogenital schistosomiasis (aOR: 2.548, 95% CI:  0.836-7.761; p=0.100) or intestinal parasites (aOR: 2.099, 95% CI: 0.973-4.521, p=0.059)  Intensity of *S. haematobium* infection (R=0.666, p=0.009) was correlated with duration of T2DM* |
| Aravindhan, 2010  (73) | Human (serum filarial antigen and IgG + IgG4 antibody), cross-sectional | India | Mixed filarial species (*W. bancrofti, Brugia malayi*) | T2DM | 1416 | Unclear (reports different proportions of women across different categories of glucose tolerance) | Unclear (reports average ages across different categories of glucose tolerance, ranging from ~38 to 51 years)* | ↓ T2DM prevalence* (10.4% of LF+ in normal glucose tolerance group, 9.1% in impaired glucose tolerance group, 5.7% in newly diagnosed T2DM group, and 4.3% in known T2DM) |
| Aravindhan, 2010  (74) | Human (serum filarial antigen and IgG + IgG4 antibody), cross-sectional | India | Mixed filarial species (*W. bancrofti, B. malayi*) | T1DM | 762 | 63.9% vs. 52.5% (normal glucose tolerance vs. T1DM group) | 35 vs. 28 years (normal glucose tolerance vs. T1DM group) | ↓ T1DM* (0% LF+ in T1DM group vs. 2.6% in the normal glucose tolerance group) |
| Chen, 2013  (25) | Human (study-defined PSI criteria), cross-sectional | China | *Schistosoma* species | Diabetes by HbA1c^#^ | 3913 | 47.1 vs. 61.4% (with PSI vs. without PSI)* | 70.5 vs. 67.6 years (with PSI vs. without PSI)* | ↓ prevalence of diabetes* (and A1c*) |
| Hays, 2015  (32) | Human (serum parasite IgG antibody), cross-sectional | Australia | *S. stercoralis* | T2DM^#^ | 259 | 59.1% | 43.4 | ↓ risk of T2DM* |
| Htun, 2018  (71) | Human (stool microscopy), cross-sectional | Laos | Mixed helminths (*A. lumbricoides, T. trichiura, S. stercoralis, O. viverrini, Taenia* spp., hookworm) | Diabetes by HbA1c | 1528 | 70.7% | Unclear (reports age ranges from 35 to 95 years) | ↑ diabetes risk (HbA1c)* in those with *Taenia* spp. infections |
| Kalantari, 2019  (90) | Human (stool microscopy, serum parasite IgG antibody); cross-sectional | Iran | *S. stercoralis* | Diabetes | 180 | 77.2% | 55.8 years | No difference  (though reported ↑ frequency of diabetes in those with positive serology (not significant; did not compare to those without DM)) |
| Li, 2016  (6) | Human (hepatosplenic disease confirmed with imaging and clinical findings + history of infection; stool microscopy used to exclude active infection); cross-sectional | China | *S. japonicum* | DM, insulin resistance^#^ | 82 | 57% | 73.7 vs. 72.6 (infected vs. controls) | ↑ prevalence of DM (37%, unclear significance) and IR* in those with chronic hepatosplenic S. japonicum and normal liver function with portal systemic shunting |
| Machado, 2018  (87) | Human (stool microscopy); cross-sectional | Brazil | Mixed helminths (*A. lumbricoides, hookworm, S. mansoni, S. stercoralis, Taenia* species) | T1DM and T2DM | 156 | 57% vs. 78% (females with T1DM vs. T2DM) | Unclear (reports age ranges from < 10 to > 81 years) | ↑ frequency of *Ascaris lumbricoides* seen in T2DM vs. T1DM* |
| McGuire, 2019  (88) | Human (parasite IgG antibody, stool microscopy and other techniques); case-control | United Kingdom | *S. stercoralis* | Diabetes | 532 | 43.4% | 48 years | ↑ frequency of diabetes* |
| Mendonca, 2006  (89) | Human (stool microscopy and techniques with serum antibody testing); case-control | Brazil | *S. stercoralis* | T2DM | 120 | 58.9% vs. 59.5% (participants with known T2DM vs. those without T2DM) | 54.1 vs. 53.9 years (participants with known T2DM vs. those without T2DM) | ↑ frequency of diabetes* |
| PrayGod, 2022  (69) | Human (stool and urine microscopy), cross-sectional | Tanzania | Mixed helminths (*S. mansoni + haematobium, A. lumbricoides, S. stercoralis, T. trichiura*) | Hemoglobin A1c, fasting glucose^#^ | 1718 | 54.8% vs. 59.2% (approx. average of infected vs. uninfected) | 39.5 vs. 40.8 years (approx. average of infected vs. uninfected) | HIV-infected:  No difference in HbA1c or fasting glucose, regardless of helminth infection or ART status  HIV-uninfected:  ↓ HbA1c* (5.2% vs. 5.5%) in those with STH vs. uninfected but not with schistosome infection; no difference in fasting glucose |
| Talukder, 2022  (68) | Human (parasite IgG antibody), cross-sectional | Australia | *S. stercoralis* | Diabetes^#^ | 536 | 55.1% vs. 54.2% (infected vs. uninfected) | 40.4 vs. 38.0 (infected vs. uninfected) | ↓ odds of diabetes* (aOR 0.58, 95% CI 0.35, 1.00; p = 0.049) but this was lost when BMI was included in the model |
| Zou, 2021  (53) | Human (study-defined PSI criteria), cross-sectional | China | *Schistosoma* species | Diabetes^#^ | 2867 | 20.7% vs. 20.3% (PSI vs. without PSI) | 68.5 vs. 68.0 years (PSI vs. without PSI) | No difference in diabetes (19.2% vs. 22.4% in those with PSI vs. without PSI, p=0.063) |
| **Animal studies (n=11)** | | | | | | | | |
| Amdare, 2017  (72) | Animal (BALB/c mice induced with STZ); recombinant Wuchereria bancrofti L2 (rWbL2) and Brugia malayi abundant larval transcript 2 (rBmALT-2)) | India | Mixed filarial species (*W. bancrofti, B. malayi*) | T1DM, serum glucose, pancreatic islet histological changes | Unclear, possibly 36-48 | 100% (only female mice used) | 6-8 weeks | ↓ incidence of T1DM by the end of the experiment (8 weeks) (by 57.2%, 66.6%, and 73.5% of diabetic mice treated with rWbL2, rBmALT-2, or a combination of the two, respectively) vs. almost all diabetic mice treated with Alum (placebo) remaining diabetic; above did not appear significant  ↓ blood glucose* and ↓ pancreatic islet inflammation and cell destruction* in mice receiving one or a combination of the proteins vs. Alum group |
| Cooke, 1999  (75) | Animal (NOD mice; cercariae) | United Kingdom | *S. mansoni* | Insulin dependent diabetes (IDDM) | Unclear, possibly 36 | 100% (only female mice used) | 6-7 vs. 5-6 week old (50 cercariae injected intraperitoneally) vs. (30 cercariae injected percutaneously) | ↓ spontaneous incidence of IDDM* in both experiments vs. PBS (control) |
| El-Gebaly, 2019  (76) | Animal (Swiss Albino miss induced with STZ; SEA) | Egypt | *S. mansoni* | T1DM | 90 | 0% (only male mice used) | 6 weeks | ↓ blood glucose* at 2- and 4 weeks in mice receiving SEA alone, SEA as curative treatment after STZ, or SEA as preventive before STZ; ↓ pancreatic inflammation and other architectural changes in STZ+SEA treatment group compared to STZ only group at 4 weeks (unclear significance) |
| El-Wakil, 2002  (77) | Animal (cercariae; C57BL/6J mice induced with STZ) | Egypt | *S. mansoni* | T1DM | 30 | 100% (only female mice used) | 8 weeks | ↓ blood glucose* and pancreatic tissue degeneration* |
| Lund, 2014  (78) | Animal (excretory/secretory parasite product, FhES); NOD/Lt mice | Unclear, possibly Australia | *Fasciola hepatica* | T1DM | Unclear | 100% (only female mice used) | 4 weeks | ↓ incidence of T1DM* (84% of mice treated with FhES vs. 19% of mice treated with PBS were protected against T1DM) with ↓ reduction in pancreatic islet inflammation* |
| Lund, 2016  (79) | Animal (recombinant parasite proteins (FhCL1 and FhHDM-1)); NOD/Lt mice | Unclear, possibly Australia | *Fasciola hepatica* | T1DM | Not reported | 100% (only female mice used) | 4 weeks | ↓ incidence of T1DM* (50% of mice treated with FhHDM-1 vs. 84% of mice treated with PBS developed diabetes) with ↓ reduction in pancreatic islet inflammation* |
| Osada, 2017  (80) | Animal (cercariae); WT C57BL/6, STAT6KO, IL-10KO, and STAT6/IL-10 double-deficient mice | Japan | *S. mansoni* | T1DM | Not reported | Not reported | 6-8 weeks | ↓ hyperglycemia* and degradation* of pancreatic islets in infected mice receiving STZ vs. uninfected mice receiving STZ |
| Reddy, 2017  (81) | Animal (recombinant filarial abundant larval protein, rBmALT-2, as therapeutic); BALB-c mice (induced with STZ) | India | *B. malayi* | T1DM | Unclear, possibly 24-32 | 100% (only female mice used) | 6-8 weeks | ↓ fasting blood glucose levels* in mice treated with rBmALT-2 vs. ALUM; 28% of diabetic mice treated with the protein became normal by end of the 2^nd^ week*, and by the end of the 5^th^ week, all became normal with reversal of pancreatic histopathological damage* |
| Wang, 2021  (82) | Animal (SEA; BALB/c and NOD mice) | China | *Schistosoma* species | T1DM | Unclear | 100% (only female mice used) | 4 weeks | ↓ incidence of diabetes* (none of the mice injected with SEA developed T1DM while 70% receiving placebo had diabetes at 25 weeks of age in NOD mice) |
| Yan, 2020  (83) | Animal (parasite recombinant enzymes, rSjcystatin and rSjFBPA); NOD mice | China | *S. japonicum* | T1DM | 30 | 100% (only female mice used) | 3 weeks | ↓ incidence of diabetes* in mice receiving either recombinant enzymes (by 40%) compared with PBS-treated mice (in controls, 100% developed diabetes) |
| Zaccone, 2003 (84) | Animal (parasite eggs, SEA, or SWA; NOD, NOD-SCID, C57BL/6 and TO mice) | United Kingdom | *S. mansoni* | T1DM | Not reported | Not reported | Not reported | ↓ incidence of diabetes* in mice exposed to eggs, SEA, or SWA (by up to 100% prevention) if injection was started at 4 weeks of age as compared to all control mice developing diabetes) |
