# Supplementary material for: Effects of helminths and anthelmintic treatment on cardiometabolic diseases and risk factors: A systematic review
Source: PLoS Negl Trop Dis. 2023 Feb 24;17(2):e0011022. doi: 10.1371/journal.pntd.0011022 (PMC9956023; doi:10.1371/journal.pntd.0011022)
Supplement: S5 Table — Abbreviations: CAD, coronary artery disease; MI, myocardial infarction; IQR, interquartile range; STH, soil-transmitted helminths; LF, lymphatic filariasis; PCR, polymerase chain reaction; PSI, previous schistosome infection; SEA, soluble egg antigen; HFD, high-fat diet; PBS, phosphate buffered saline. #study investigated other outcome measures that will be included in other tables. *denotes statistical significance, p<0.05. (DOCX) [file pntd.0011022.s005.docx]

| **Overview:**   - 6 animal and 5 human studies - None investigated the subsequent effects of anthelmintic treatment on CAD, MI, or atherosclerosis - Estimated median sample size = 319 [IQR 40-675] - Helminths represented: *S. mansoni* (5), unspecified *Schistosoma* species (2), *S.* *japonicum* (1), mixed filarial species (1), mixed STH (1), and *Opisthorchis* species (1) - Human studies:   - 3 cross-sectional and 2 cadaver studies  - Median age: 51.5 years [IQR 45-68.5]  - Median percent of women: 27.6% [IQR 16.5-48.4]   - Animal studies:   - All mouse studies  - 2 studies clearly reported sex—all male mice were used (other studies were unclear in reporting) | | | | | | | | |
| --- | --- | --- | --- | --- | --- | --- | --- | --- |
| **Study, Year (reference #)** | **Study type (animal model, method of infection/diagnosis)** | **Country** | **Parasite Species** | **Outcome** | **Sample Size** | **Sex (% Female)** | **Age in Years (Mean or Median)** | **Effect of Parasite and Anthelmintic Treatment on Outcome** |
| **Studies examining CAD, MI, or atherosclerosis only cross-sectionally (n=11)** | | | | | | | | |
| **Human studies (n=5)** | | | | | | | | |
| Aravindhan, 2012  (22) | Human (serum filarial antigen and IgG + IgG4 antibody), cross-sectional | India | Mixed filarial species (*W. bancrofti* and *B. malayi*) | CAD, carotid intimal thickness^#^ | 453 | 34.5% | 51.5 vs. 51.7 years (CAD+ group vs. CAD- group) | No association between LF and CAD prevalence  No difference in carotid intimal thickness between LF+ and LF- individuals in the CAD+ group |
| Magen, 2013  (38) | Human cadaver/autopsy (liver examination for worms) | Russia | *Opisthorchis* species | Aortic atherosclerosis^#^ | 319 | 12.2% | Unclear (reports age ranges from 20 to > 60 years) | ↓ aortic atherosclerosis* (magnified with increasing infection burden) across all age ranges |
| Shalaby, 1963  (91) | Human cadaver/autopsy (unclear criteria for diagnosis of infection—per study, there were “cases of clinically diagnosed bilharzial hepatosplenomegaly”) | Egypt | *Schistosoma* species | Myocardial infarction, atherosclerosis^#^ | 571 autopsies but only 54 cases of bilharzial cirrhosis | Not reported | Not reported | ↓ MI* and atherosclerosis* frequencies in autopsies with bilharzial cirrhosis compared to all other autopsies (? but no different from other cases of cirrhosis) |
| Wiria, 2013  (47) | Human (stool microscopy with stool PCR), cross-sectional | Indonesia | Mixed helminths (*T. trichiura, A. lumbricoides, N. americanus, A. duodenale, S. stercoralis*) | Carotid intimal thickness^#^ | 675 | 62.3% vs. 65.9% (infected vs. uninfected) | 45.0 vs. 44.8 years (infected vs. uninfected) | No difference in carotid intimal thickness |
| Zou, 2021  (53) | Human (study-defined PSI criteria), cross-sectional | China | *Schistosoma* species | CAD^#^ | 2867 | 20.7% vs. 20.3% (PSI vs. without PSI) | 68.5 vs. 68.0 years (PSI vs. without PSI) | ↓ prevalence of CAD* (unadjusted regression OR 1.8 (95% CI, 1.16-2.79); p=0.008; remained significant even after adjusting for a number of laboratory measures and hepatic dysfunction) |
| **Animal studies (n=6)** | | | | | | | | |
| Cortes-Selva, 2018  (61) | Animal (C57BL/6 and ApoE-deficient mice; cercariae) | United States | *S. mansoni* | Aortic atherosclerosis^#^ | Unclear | Unclear, but possibly only male mice used | Unclear, possibly 6 weeks of age | ↓ aortic plaque area* |
| Doenhoff, 2002  (27) | Animal (ApoE-deficient and random-bred TO mice; cercariae) | United Kingdom | *S. mansoni* | Atherosclerotic lesions in aortic arch and brachiocephalic artery^#^ | Unclear, possibly 71 | Unclear, possibly 52.1% | Not reported | ↓ atherosclerotic lesions* in infected ApoE-deficient mice by 50% |
| LaFlamme, 2007  (35) | Animal (ApoE-deficient C57BL/6 mice; parasite egg exposure) | New Zealand | *S. mansoni* | Aortic atherosclerosis^#^ | Unclear | Not reported | 3-5 weeks for atherosclerosis studies | No difference in atherosclerotic lesion size |
| Toulah, 2018  (46) | Animal (Swiss albino mice; SEA or cercariae) | Egypt | *S. mansoni* | Arterial resistance, blood flow velocity, aortic arch atherosclerosis^#^ | 40 | 0% (only male mice used) | 6-8 weeks | ↓ arterial resistance*, ↓ aortic atherosclerosis (unclear significance), and ↑ blood flow velocity* in mice on HFD exposed to either SEA or cercariae (though SEA exposed mice showed more improved architectural changes) |
| Wolfs, 2014  (49) | Animal (C57BL/6 wild-type and LDL R^-/-^ mice; SEA) | The Netherlands | *S. mansoni* | Aortic root atherosclerosis^#^ | Unclear, possibly 20-40 | Not reported | Not reported | ↓ aortic plaque size* and progression* by 44% in LDLR^-/-^ mice on HFD exposed to SEA vs. PBS (control) |
| Yang, 2021  (51) | Animal (ApoE-deficient C57BL/6 mice; parasite recombinant protein, rSj-Cys) | China | *S. japonicum* | Aortic atherosclerosis, kidney/glomerular damage and fat deposition, blood flow velocity^#^ | 24 | 0% (only male mice used) | 7-8 weeks old | ↓ atherosclerotic plaque on entire aorta* and aortic sinus*, ↓ fat deposition in kidney* and ↓ glomerular damage*, and improved blood flow of the heart (↓ peak velocity* and mean gradient* of ascending aorta) associated with exposure to rSj-Cys in mice fed HFD vs. unexposed mice fed HFD |
