# Supplementary material for: Effects of helminths and anthelmintic treatment on cardiometabolic diseases and risk factors: A systematic review
Source: PLoS Negl Trop Dis. 2023 Feb 24;17(2):e0011022. doi: 10.1371/journal.pntd.0011022 (PMC9956023; doi:10.1371/journal.pntd.0011022)
Supplement: S6 Table — Abbreviations: IQR, interquartile range; STH, soil-transmitted helminths; SBP, systolic blood pressure; DBP, diastolic blood pressure; PZQ, praziquantel; PCR, polymerase chain reaction; RCT, randomized controlled trial; BP, blood pressure; PSI, previous schistosome infection; MAP, mean arterial pressure; PVR, peripheral vascular resistance; #study investigated other outcome measures that will be included in other tables. *denotes statistical significance, p<0.05. (DOCX) [file pntd.0011022.s006.docx]

| **Overview:**   - 17 human studies and 1 animal study - Estimated median sample size = 555 [IQR 181-1898] - Helminths represented: unspecified *Schistosoma* species (6), mixed STH (5), *O. viverrini* (2), *S. mansoni* (2), *S. stercoralis* (2)*,* and *O. volvulus* (1) - Only 3 of 18 studies reported on both baseline and follow-up blood pressure and cardiovascular hemodynamics (before and after anthelmintic treatment) - Human studies:   - 13 cross-sectional, 2 randomized clinical trials, 1 prospective cohort, and 1 cadaver study  - Median age: 44.6 years [IQR 41.5-65.4]  - Median percent of women: 48% [IQR 27.7-60]   - Animal studies:   - 1 mouse study; sex not reported | | | | | | | | |
| --- | --- | --- | --- | --- | --- | --- | --- | --- |
| **Study, Year (reference #)** | **Study type (animal model, method of infection/diagnosis)** | **Country** | **Parasite Species** | **Outcome** | **Sample Size** | **Sex (% Female)** | **Age in Years (Mean or Median)** | **Effect of Parasite and Anthelmintic Treatment on Outcome** |
| **Studies examining blood pressure and cardiovascular hemodynamics before and after anthelmintic treatment (n=3)** | | | | | | | | |
| **Human studies (n=3)** | | | | | | | | |
| Muthukumar, 2020  (14) | Human (stool microscopy, prospective cohort | Thailand | *O. viverrini* | SBP and DBP^#^ | 400 | 60% | Unclear  (age≤50: n=219; age≥51: n=181) | Baseline  No difference in SBP or DBP  Follow-up  No effect on SBP or DBP in infected individuals (6 months after PZQ) |
| Sanya, 2020  (13) | Human (stool microscopy and PCR), cluster-RCT | Uganda | Mixed helminths (*S. mansoni, S. stercoralis, T. trichiura*) | SBP and DBP^#^ | 1898 | 46.5% vs. 47.1% (intensive vs. standard anthelmintic treatment) | 32 vs. 31 years (intensive vs. standard anthelmintic treatment) | Baseline  ↓DBP* in those with heavy *S. mansoni* infection  Follow-up  No effect on BP in either intensive or standard anthelmintic treatment arms (PZQ/albendazole) after 4 years of follow-up |
| Tahapary, 2017  (8) | Human (stool microscopy, stool PCR), cluster-RCT | Indonesia | Mixed helminths (*A. lumbricoides, T. trichiura, S. stercoralis*) | SBP and DBP^#^ | 1669 | 60% vs. 61.2% (albendazole treatment vs. placebo) | 42.5 vs. 42.5 years (albendazole treatment vs. placebo) | Baseline  No difference in SBP or DBP (albendazole vs. control)  Follow-up  No effect on SBP or DBP after 52 weeks of follow-up |
| **Studies examining blood pressure and cardiovascular hemodynamics only cross-sectionally (n=15)** | | | | | | | | |
| **Human studies (n=14)** | | | | | | | | |
| Buck, 1971  (23) | Human (urine microscopy), cross-sectional | Republic of Chad | *O. volvulus* | SBP and DBP^#^ | 153 | 48% | Unclear (reports age ranges from 0 to > 40 years) | ↓ SBP* (t=2.7); no difference in DBP |
| Changbumrung, 1988  (24) | Human (stool microscopy), cross-sectional | Thailand | *O. viverrini* | Blood pressure (not specified)^#^ | 42 | Not reported | Unclear (reports age ranges from 14-69 years) | No difference in blood pressure |
| Chen, 2013  (25) | Human (study-defined PSI criteria), cross-sectional | China | *Schistosoma* species | SBP and DBP^#^ | 3913 | 47.1 vs. 61.4% (with PSI vs. without PSI)* | 70.5 vs. 67.6 years (with PSI vs. without PSI)* | No difference in SBP or DBP |
| Gonzalez-Fernandez, 2020  (92) | Human (stool microscopy), cross-sectional | Panama | Mixed helminths (hookworm, *Ascaris, Trichuris*) | Maternal blood pressure | 213 | 100% (only pregnant women enrolled) | Unclear (reports categories of age ranges) | Hookworm:↑ SBP*, DBP*, MAP*  Trichuris: ↑ odds of elevated MAP* |
| Hays, 2015  (32) | Human (parasite IgG antibody), cross-sectional | Australia | *S. stercoralis* | SBP and DBP^#^ | 259 | 59.1% | 43.4 | No clear difference in SBP or DBP  (↑ SBP* in diabetics compared to non-diabetics* but no difference in DBP—however, no clear comparison of blood pressure between infected and uninfected) |
| Mohamed, 2017  (56) | Human (stool microscopy to rule out active infection; study-defined PSI criteria), cross-sectional | Egypt | *Schistosoma* species | SBP and DBP^#^ | 574 | 27.7% | 56.7 vs, 57.9 (infected vs. uninfected) | ↓SBP* and DBP* |
| Sanya, 2020  (7) | Human (stool microscopy, stool PCR), cross-sectional | Uganda | Mixed helminths (*S. mansoni, T. trichiura, A. lumbricoides, S. stercoralis*) | SBP and DBP ^#^ | 2828 | 49% vs. 65% (rural vs. urban survey)* | 31.5 vs. 29.7 years (rural vs. urban survey)* | No clear difference in SBP or DBP  (↑ SBP and DBP in urban residents*, but current helminth infection did not explain differences) |
| Shalaby, 1963  (91) | Human cadaver/autopsy (unspecified clinical criteria for diagnosis of infection) | Egypt | *Schistosoma* species | Hypertension^#^ | 571 autopsies but only 54 cases of bilharzial cirrhosis | Not reported | Not reported | ↓ hypertension* frequency in autopsies with bilharzial cirrhosis compared to all other autopsies (? unclear if different from other cases of cirrhosis) |
| Shen, 2014  (43) | Human (study-defined PSI criteria), cross-sectional | China | *Schistosoma* species | SBP and DBP^#^ | 1942 | With PSI: 11.6%  Without PSI: 20.1% | 65.7 vs. 64.9 years (men with PSI vs. without PSI)  64.4 vs. 65.4 years (women with PSI vs. without PSI) | No differences in hypertension between men and women with or without PSI  ↓ DBP* but no difference in SBP in men with PSI vs. men without PSI |
| Shen, 2015  (42) | Human (study-defined PSI criteria + stool microscopy used to exclude active infection), cross-sectional | China | *Schistosoma* species | SBP and DBP^#^ | 1597 | None (only men enrolled) | 65.7 vs. 64.9 years (with PSI vs. without PSI) | ↓ hypertension* in those with PSI, before adjustment for age, BMI, and other factors  ↓ DBP* among those with PSI but no difference in SBP |
| Talukder, 2022  (68) | Human (parasite IgG antibody), cross-sectional | Australia | *S. stercoralis* | Hypertension^#^ | 536 | 55.1% vs. 54.2% (infected vs. uninfected) | 40.4 vs. 38.0 (infected vs. uninfected) | No difference in hypertension |
| Wiria, 2013  (47) | Human (stool microscopy with stool PCR), cross-sectional | Indonesia | Mixed helminths (*T. trichiura, A. lumbricoides, N. americanus, A. duodenale, S. stercoralis*) | SBP and DBP^#^ | 675 | 62.3% vs. 65.9% (infected vs. uninfected) | 45.0 vs. 44.8 years (infected vs. uninfected) | No differences in SBP or DBP |
| Wolde, 2019  (48) | Human (stool microscopy), cross-sectional | Ethiopia | *S. mansoni* | SBP and DBP^#^ | 181 | *S. mansoni* positive (endemic): 41.5%  *S. mansoni* negative (endemic): 49.4%  *S. mansoni* negative (non-endemic): 29.5% | *S. mansoni* positive (endemic): 44.2  *S. mansoni* negative (endemic): 39.9  *S. mansoni* negative (non-endemic): 28.1 | No differences in SBP or DBP in either endemic or non-endemic region |
| Zou, 2021  (53) | Human (study-defined PSI criteria), cross-sectional | China | *Schistosoma* species | Hypertension^#^ | 2867 | 20.7% vs. 20.3% (PSI vs. without PSI) | 68.5 vs. 68.0 years (PSI vs. without PSI) | No difference in hypertension |
| **Animal studies (n=1)** | | | | | | | | |
| Sarin, 1991  (93) | Animal (C3H mouse; cercariae) | United States | *S. mansoni* | MAP and PVR | 19 | Not reported | 5- to 7-week-old | ↓ MAP* and PVR* |
