# Supplementary material for: Effects of helminths and anthelmintic treatment on cardiometabolic diseases and risk factors: A systematic review
Source: PLoS Negl Trop Dis. 2023 Feb 24;17(2):e0011022. doi: 10.1371/journal.pntd.0011022 (PMC9956023; doi:10.1371/journal.pntd.0011022)
Supplement: S7 Table — Abbreviations: hsCRP, high-sensitivity C-reactive protein; IQR, interquartile range; STH, soil-transmitted helminths; PCR, polymerase chain reaction; RCT, randomized controlled trial; LF, lymphatic filariasis; CAD, coronary artery disease. #study investigated other outcome measures that will be included in other tables. *denotes statistical significance, p<0.05. (DOCX) [file pntd.0011022.s007.docx]

| **Overview:**   - All 5 studies investigating hsCRP were human studies - Estimated median sample size = 646 [IQR 453-675] - Helminths represented: mixed STH (3), mixed filarial species (1), *S. haematobium* (1) - 4 cross-sectional studies and 1 randomized clinical trial - Median age: 45 [IQR 42.5-45.2] - Median percent of women: 60% [IQR 51.3-62] | | | | | | | | |
| --- | --- | --- | --- | --- | --- | --- | --- | --- |
| **Study, Year (reference #)** | **Study type (animal model, method of infection/diagnosis)** | **Country** | **Parasite Species** | **Outcome** | **Sample Size** | **Sex (% Female)** | **Age in Years (Mean or Median)** | **Effect of Parasite and Anthelmintic Treatment on Outcome** |
| **Studies examining high-sensitivity C-reactive protein before and after anthelmintic treatment (n=1)** | | | | | | | | |
| **Human studies (n=1)** | | | | | | | | |
| Tahapary, 2017  (8) | Human (stool microscopy, stool PCR), cluster-RCT | Indonesia | Mixed helminths (*A. lumbricoides, T. trichiura, S. stercoralis*) | hsCRP^#^ | 1669 | 60% vs. 61.2% (albendazole treatment vs. placebo) | 42.5 vs. 42.5 years (albendazole treatment vs. placebo) | Baseline  No difference in hsCRP between the albendazole vs. placebo group  Follow-up  No effect after 52 weeks of follow-up |
| **Studies examining high-sensitivity C-reactive protein only cross-sectionally (n=4)** | | | | | | | | |
| **Human studies (n=4)** | | | | | | | | |
| Aravindhan, 2012  (22) | Human (serum filarial antigen and IgG + IgG4 antibody), cross-sectional | India | Mixed filarial species (*W. bancrofti* and *B. malayi*) | hsCRP^#^ | 453 | 34.5% | 51.5 vs. 51.7 years (CAD+ group vs. CAD- group) | No difference in hsCRP between LF+ and LF- individuals within CAD+ group  ↑ hsCRP in the CAD+ group vs. CAD- group* in general |
| Wiria, 2013  (47) | Human (stool microscopy with stool PCR), cross-sectional | Indonesia | Mixed helminths (*T. trichiura, A. lumbricoides, N. americanus, A. duodenale, S. stercoralis*) | hsCRP^#^ | 675 | 62.3% vs. 65.9% (infected vs. uninfected) | 45.0 vs. 44.8 years (infected vs. uninfected) | No difference in hsCRP |
| Wiria, 2015  (65) | Human (stool microscopy with stool PCR), cross-sectional | Indonesia | Mixed helminths (*T. trichiura, A. lumbricoides, N. americanus, A. duodenale, S. stercoralis*) | hsCRP^#^ | 646 | 62.0% vs. 66.2% (infected vs. uninfected) | 45.2 vs. 44.4 years (infected vs. uninfected) | No difference in hsCRP |
| Zinsou, 2020  (52) | Human (urine microscopy), cross-sectional | Gabon | *S. haematobium* | hsCRP^#^ | 71 | 51.3% vs. 56.2%. (infected vs. uninfected) | 34.5 vs. 35.7 years (infected vs. uninfected) | No difference in hsCRP |
