# Supplementary material for: Effects of helminths and anthelmintic treatment on cardiometabolic diseases and risk factors: A systematic review
Source: PLoS Negl Trop Dis. 2023 Feb 24;17(2):e0011022. doi: 10.1371/journal.pntd.0011022 (PMC9956023; doi:10.1371/journal.pntd.0011022)
Supplement: S8 Table — Abbreviations: IQR, interquartile range; STH, soil-transmitted helminths; HFD, high-fat diet; SD, standard diet; SEA, soluble egg antigen. #study investigated other outcome measures that will be included in other tables. *denotes statistical significance, p<0.05. (DOCX) [file pntd.0011022.s008.docx]

| **Overview:**   - 2 human and 2 animal studies - None investigated the subsequent effects of anthelmintic treatment on non-atherosclerotic cardiovascular disease - Estimated median sample size = 45 [IQR 30-80] - Helminths represented: *S. mansoni* (2), mixed STH (1), and *Opisthorchis* species (1) - Human studies:   - 1 cross-sectional and 1 cadaver study  - Median age: 38.7 years [IQR 25-52.3]  - Median percent of women: only 1 study clearly reported sex   - Animal studies:   - 2 mouse studies   - - 1 used all male and the other all female mice | | | | | | | | |
| --- | --- | --- | --- | --- | --- | --- | --- | --- |
| **Study, Year** | **Study type (animal model, method of infection/diagnosis)** | **Country** | **Parasite Species** | **Outcome** | **Sample Size** | **Sex (% Female)** | **Age in Years (Mean or Median)** | **Effect of Parasite and Anthelmintic Treatment on Outcome** |
| **Studies examining non-atherosclerotic cardiovascular disease only cross-sectionally (n=4)** | | | | | | | | |
| **Human studies (n=2)** | | | | | | | | |
| Bychkov, 2019  (96) | Human cadaver/autopsy (unclear criteria for diagnosis of infection—per study, cases were from “healthcare facilities treating opisthorchiasis patients”) | Russia | *Opisthorchis* species | Eosinophilic myocarditis, hypereosinophilic syndrome | 110 | Unclear (there were 361 females in the 578 cases, but only 110 individuals were subjected to morphological/pathological study) | 52.3 years (autopsy cases) | ↑ associated cardiac changes, including eosinophilic myocarditis, in those with invasive opisthorchiasis  (unclear statistical significance; unclear control group) |
| Carranza-Rodriguez, 2017  (97) | Human (stool microscopy and other stool tests/techniques; urine microscopy; blood smear for microfilariae; serological antigen tests) cross-sectional | Spain | Mixed helminths (*S. stercoralis, Wuchereria bancrofti, Schistosoma*, and *Fasciola* species, as well others) | Endomyocardial clinical manifestations of eosinophilia | 50 | 6% | 25.0 vs. 26.0 years (study group, eosinophilia > 450 cells/μ  L vs. control group, eosinophilia <450 cells/μ  L | No difference in endomyocardial changes  (Significant association between presence of eosinophilia and cardiac valvular alterations (posterior mitral leaflet thickening)*, but no association between degree of eosinophilia or type of parasite and cardiac valve involvement) |
| **Animal studies (n=2)** | | | | | | | | |
| Góes, 2012  (98) | Animal (Swiss Webster mice; cercariae) | Brazil | *S. mansoni* | Myocardial injury | 20 | 100% (only female mice were used) | Not reported | Acute infection (9 weeks): ↓ density of cardiomyocytes*, ↑ area of injury* showing more myocarditis, and ↑ collagen* (all infected vs. uninfected mice fed either diet).  Chronic infection (17 weeks): ↓ total cardiomyocytes* (all infected vs. uninfected mice fed either diet) and ↑ area of injury* showing more myocarditis (infected HFD vs. infected SD mice) |
| Toulah, 2018  (46) | Animal (Swiss albino mice; SEA or cercariae) | Egypt | *S. mansoni* | Myocardium lipid deposits^#^ | 40 | 0% (only male mice used) | 6-8 weeks | ↓ myocardial lipid deposits and cardiac hypertrophy (both of unclear significance) in mice on HFD exposed to either SEA or cercariae (though SEA exposed mice showed more improved architectural changes) |
