# Supplementary material for: Effects of helminths and anthelmintic treatment on cardiometabolic diseases and risk factors: A systematic review
Source: PLoS Negl Trop Dis. 2023 Feb 24;17(2):e0011022. doi: 10.1371/journal.pntd.0011022 (PMC9956023; doi:10.1371/journal.pntd.0011022)
Supplement: S9 Table — (DOCX) [file pntd.0011022.s009.docx]

| **Cardiometabolic disease or risk factor** | **Number of studies** | **Median sample size** | **Overall effect of helminths on outcome** | **Overall effect of anthelmintic treatment on outcome** |
| --- | --- | --- | --- | --- |
| Serum lipids | 45 | 167.5 | Mostly ↓ (36 of 45 studies) | Mostly ↑ (5 of 6 studies) |
| Metabolic syndrome^*^ | 38 | 213.5 | Mostly ↓ (22 of 38 studies) | Mostly ↑ (7 of 10 studies) |
| Diabetes | 30 | 279.5 | Mostly ↓ (17 of 29 studies) | Mostly ↑ (3 of 5 studies) |
| Atherosclerotic cardiovascular disease | 11 | 319 | -Atherosclerosis: Mostly ↓ (7 of 10 studies)  -CAD/MI: Mostly ↓ (2 of 3 studies) | Not studied |
| Blood pressure | 18 | 555 | Mixed (no effect in 10 of 18 studies) | No effect (3 of 3 studies) |
| High-sensitivity CRP | 5 | 646 | No effect (5 of 5 studies) | No effect (1 of 1 study) |
| Non-atherosclerotic cardiovascular disease | 4 | 45 | Mixed (2 showed ↑, 1 showed ↓, and 1 reported no effect) | Not studied |
