# Supplementary material for: Effects of helminths and anthelmintic treatment on cardiometabolic diseases and risk factors: A systematic review
Source: PLoS Negl Trop Dis. 2023 Feb 24;17(2):e0011022. doi: 10.1371/journal.pntd.0011022 (PMC9956023; doi:10.1371/journal.pntd.0011022)
Supplement: S1 Supplemental Table — (DOCX) [file pntd.0011022.s010.docx]

| # | First Author, Year, Journal | Downs and Black Total Score  (out of 28 total possible points) |
| --- | --- | --- |
| 1 | Assaadkhalil, 1992, Annales De Biologie Clinique | 13 |
| 2 | Ata, 1958, Zeitschrift fur Tropenmedizin und Parasitologie | 8 |
| 3 | Bastos, 1985, Revista de saude publica | 12 |
| 4 | Bibawi, 1970, Transactions of the Royal Society of Tropical Medicine and Hygiene | 8 |
| 5 | Carlisle, 1972, British Heart Journal | 13 |
| 6 | Chaidee, 2016, American Journal of Tropical Medicine and Hygiene | 9 |
| 7 | Coutinho-Abath, 1965, Revista brasileira de malariologia e doencas tropicais | 10 |
| 8 | Dimenstein, 1992, Brazilian Journal of Medical and Biological Research | 12 |
| 9 | el-Badry, 1976, The Journal of the Egyptian Medical Association | 11 |
| 10 | Farias, 1997, Hypertension | 5 |
| 11 | Gabr, 1973, Acta biologica et medica Germanica | 10 |
| 12 | Gad-el-Mawla, 1972, The Journal of the Egyptian Medical Association | 10 |
| 13 | Ghanem, 1970, Atherosclerosis | 10 |
| 14 | Ghanem, 1971, Journal of Tropical Medicine and Hygiene | 11 |
| 15 | Gillett. 1976, Lancet | 12 |
| 16 | Guimaraes, 2009, Revista do Colégio Brasileiro de Cirurgiões | 10 |
| 17 | Kaewpitoon, 2016, Asian Pacific Journal of Cancer Prevention | 5 |
| 18 | Mansour, 1988, Journal of the Egyptian Society of Parasitology | 10 |
| 19 | Mousa, 1975, Egyptian Journal of Bilharziasis | 12 |
| 20 | Nofal, 1973, The Journal of Tropical Medicine and Hygiene | 10 |
| 21 | Osman, 1995, Journal of the Egyptian Society of Parasitology | 8 |
| 22 | Pricoli, 1981, Arquivos de Gastroenterologia | 9 |
| 23 | Pugh, 1979, Annals of Tropical Medicine and Parasitology | 6 |
| 24 | Ryoji, 1997, The Southeast Asian Journal of Tropical Medicine and Public Health | 7 |
| 25 | Singh, 1971, The Indian Journal of Medical Research | 10 |
| 26 | Sobh, 1993, Experimental Nephrology | 10 |
| 27 | Soliman, 1996, Journal of Tropical Pediatrics | 11 |
| 28 | Sukkar, 1974, Transactions of the Royal Society of Tropical Medicine and Hygiene | 9 |
| 29 | Sun, 2014, Chinese Journal of Microbiology and Immunology | 13 |
| 30 | Tenaguem, 2010, American Journal of Tropical Medicine and Hygiene | 12 |
| 31 | Wiedermann, 1991, [Zentralblatt für Bakteriologie](https://www.sciencedirect.com/journal/zentralblatt-fur-bakteriologie) | 11 |
| 32 | Wilkins, 1977, Annals of Tropical Medicine and Parasitology | 11 |
| 33 | Zaki, 1980, Hepato-Gastroenterology | 11 |
| 34 | Zhang, 2012, Chinese Journal of Schistosomiasis Control | 13 |
