# Supplementary material for: Effects of helminths and anthelmintic treatment on cardiometabolic diseases and risk factors: A systematic review
Source: PLoS Negl Trop Dis. 2023 Feb 24;17(2):e0011022. doi: 10.1371/journal.pntd.0011022 (PMC9956023; doi:10.1371/journal.pntd.0011022)
Supplement: S1 Appendix — (DOCX) [file pntd.0011022.s012.docx]

Ovid MEDLINE(R) ALL 1946 to December 08, 2020

Dec 10, 2020*

2581

Helminths Medline 2000_2581.ris x

Helminths Medline 1_1999.ris x

Ovid Embase 1974 to 2020 December 09

Dec 10, 2020*

4311

Helminths Embase 4000_4311.ris x

Helminths Embase 2000_3999.ris x

Helminths Embase 1_1999.ris x

Cochrane Library

Dec 10, 2020*

Cochrane Central Register of Controlled Trials: 162

Cochrane Database of Systematic Reviews: 12

Helminths Cochrane Trials 1_162.ris x

Helminths Cochrane Reviews 1_12.ris x

**Search**:

See: WOS & Cochrane Search Dec 10.doc

Web of Science Core Collection

Dec 10, 2020*

3245

KP Helminths Endnote WoS Results RIS.txt x

**Search**:

See: WOS & Cochrane Search Dec 10.doc

Global Index Medicus

Dec 10, 2020*

141

Helminths Global Index Medicus 1_141.ris x

**Amended Search:**

(tw:(Ascaris lumbricoides OR Trichuris OR Necator americanus OR Ancylostoma OR Strongyloides stercoralis OR Schistosomatidae OR Schistosoma haematobium OR Schistosoma japonicum OR Schistosoma mansoni)) OR (tw:(Wuchereria bancrofti OR Brugia malayus OR Onchocerca volvulus OR Loa OR Dracunculus OR Nematode OR Clonorchis sinensis OR Opisthorchis OR Paragonimus OR Paragonimus westermani OR Fasciolidae OR Fasciola hepatica OR Taenia solium)) OR (tw:(Ascariasis Trichuriasis OR Parasitic Helminthiasis OR Nematode Infections OR Hookworm Infections OR Ancylostomiasis OR Necatoriasis OR Strongyloidiasis OR Schistosomiasis OR Schistosomiasis haematobia OR Schistosomiasis mansoni OR Schistosomiasis japonica OR Elephantiasis OR Onchocerciasis OR Loiasis OR Dracunculiasis OR Clonorchiasis OR Opisthorchiasis OR Paragonimiasis OR Trematode Infections OR Fascioliasis Taeniasis))
