# Supplementary material for: Effects of helminths and anthelmintic treatment on cardiometabolic diseases and risk factors: A systematic review
Source: PLoS Negl Trop Dis. 2023 Feb 24;17(2):e0011022. doi: 10.1371/journal.pntd.0011022 (PMC9956023; doi:10.1371/journal.pntd.0011022)
Supplement: Attachment — Submitted filename: PLOS NTDs review notes_12sep22.docx [file pntd.0011022.s014.docx]

In this manuscript, Pham and colleagues present findings of a systematic review investigating the effects of helminth infection and anthelminthic treatment on cardiometabolic diseases and risk factors. They analysed eighty-three human and animal studies. These studies investigated the impact on lipids, metabolic syndrome, diabetes, blood pressure, atherosclerotic cardiovascular disease, high-sensitivity C-reactive protein, and non-atherosclerotic cardiovascular disease. The review shows that helminth infection may protect against cardiometabolic diseases and anthelminthic treatment may unravel this protection. These are very important findings for the discipline investigating the link between helminths and non-communicable diseases. The review is comprehensive covering both animal and human studies. Here are my concerns.

The authors need to revise the claim about the novelty of this review. There have been prior systematic reviews investigating the impact of helminths on metabolic outcomes such as Tracey et al *Do worms protect against the metabolic syndrome? A systematic review and meta-analysis* (DOI: 10.1016/j.diabres.2016.08.014) and Rennie et al *The Impact of Helminth Infection on the Incidence of Metabolic Syndrome: A Systematic Review and Meta-Analysis* (DOI: 10.3389/fendo.2021.728396). These should at least be cited or acknowledged either in the introduction or discussion of the review.

The authors have done a commendable job of searching the literature. It would be nice to state, in the manuscript, the particular time frame covered by the review Were the databases searched from inception? This is mentioned in the PROSPERO registration but not in the manuscript.

For the PRISMA flow diagram, the authors should be more explicit about the exclusion of articles based on the eligibility criteria. Can the authors provide a breakdown of the particular number of articles exclude for each exclusion criterion, especially for the full-text articles?

Also limiting the search to the major helminths has resulted in the exclusion of important work done with other helminth species. For example the impact of *Nippostrongylus brasilie*nsis on serum cholesterol (Farid et al 2010 DOI 10.1111/j.1365-2362.2010.02352.x.0, ES-62, a product from the filarial nematode *Acanthocheilonema viteae* and atherosclerosis (Aprahamian et al., 2015 doi: 10.1016/j.ijpara.2014.12.006.) and the effect of ivermectin treatment of the nematode *Metastrongylus* spp. on triglyceride levels (Lopez-Olvera et al., 2006 doi: 10.1007/s00436-005-0099-2.) This should be acknowledged. Furthermore, the review seems to have missed important early contributions on the impact of Schistosoma mansoni on lipids such as de Oliveira et al. 1977 (doi: 10.1016/0014-4894(77)90029-7) and Baumheuer et al. 1994 (Trop Med Parasitol. 1994 Mar;45(1):5-8). The authors should address that.

Please report the animal and human studies separately, preferably with sub-headings, to clearly bring out the evidence. It will make it easier for the reader to decipher that the protective link between helminths and cardiometabolic outcomes has been demonstrated more clearly in animal studies than in human studies.

As part of the reporting guidelines for systematic reviews, the authors should include a PRISMA checklist as supplementary material.

Overall, this systematic review has important findings and is well-written. I would recommend the manuscript for publication after the comments above are addressed.
