# Supplementary material for: Effects of helminths and anthelmintic treatment on cardiometabolic diseases and risk factors: A systematic review
Source: PLoS Negl Trop Dis. 2023 Feb 24;17(2):e0011022. doi: 10.1371/journal.pntd.0011022 (PMC9956023; doi:10.1371/journal.pntd.0011022)
Supplement: Attachment — Submitted filename: PLOS NTD SR--Response to Reviewers_FINAL.docx [file pntd.0011022.s015.docx]

September 20, 2022

Dear Editorial Board:

We write to submit revisions to our original manuscript titled, “Effects of Helminths and Anthelmintic Treatment on Cardiometabolic Diseases and Risk Factors: A Systematic Review”, for consideration for publication as a systematic review in *PLOS Neglected Tropical Diseases.* Our manuscript number is PNTD-D-22-00873.

We thank the Editorial Board, including Drs. Rosa and Fujiwara, and peer reviewers for considering our work and for the thoughtful comments. We respond to the comments point-by-point below and have also tracked changes in the manuscript. We appreciate the opportunity to strengthen our manuscript with these revisions.

**Reviewer # 1’s Comments:**

Comment #1: This manuscript aims to provide a systematic assessment of the causal relationship between helminth infection and cardiometabolic diseases and the effect of helminth eradication on cardiometabolic risk. The analysis of 83 papers led to the conclusion that helminth infection may offer protection against dyslipidemia, metabolic syndrome, diabetes, and atherosclerotic cardiovascular disease, and that this protection may lessen after anthelmintic treatment.

A standard approach for the selection of manuscripts for the systematic review was adopted – however, it seems that only a small number of parasites were included in the search criteria. While this was justified by the authors through the claim that these were the top ten most prevalent in human infections, I think this is an important fact, which may have skewed the collection of research data. Therefore, I think this selection approach should be clearly stated in the abstract of the manuscript.

**Response to Comment #1:** We thank the reviewer for his/her consideration of our work and for pointing out that we should add our search criteria for included helminths in the abstract of the manuscript. We have now added the selection approach of included helminths in the abstract (lines 34-35). We have also included this as a potential limitation of our review (lines 1032-1035).

Comment #2: For each medical criteria a comprehensive comparison has been made with the effect of helminth infection. However, in terms of assessing the effect of helminth infection, I would prefer to see some delineation between the animal and human studies – these should not be grouped as a collective for the determination of efficacy of helminth infection. The animal studies are all experimental infections which typically use higher doses than are ever found in endemic human single infections. Furthermore, the infection progression is more defined in the animal studies, whereas the human population are deemed infected in this review if they present with a positive antigen test – although this does not mean that they are actually actively infected.

**Response to Comment #2:** We greatly appreciate the reviewer’s comment regarding the need to better delineate between animal and human studies. As also pointed out by reviewer # 3, we agree that the animal and human studies should be reported separately to clearly bring out the evidence. We have now included subheadings in our manuscript to delineate between animal and human studies in sections pertaining to studies examining a particular outcome before and after anthelmintic therapy, where appropriate and applicable.

Most of the other sections (e.g., atherosclerotic cardiovascular disease, blood pressure and cardiovascular hemodynamics, hsCRP, and non-atherosclerotic cardiovascular disease) were already separated by human vs. animal study, and we believe that further adding subheadings may affect the flow of the manuscript. Similarly, for the cross-sectional studies in the other 3 sections (e.g., lipids, metabolic syndrome, and diabetes), we have now included the number of human vs. animal studies in parentheses where it was important to delineate between the studies. For all cross-sectional and prospective or other studies, we have also organized Tables 2-8 to newly separate human and animal studies, so that readers can quickly glance at these tables as they read through the manuscript. We have additionally added the reference numbers for every study in Tables 2-8 to further assist the reader with matching the cited studies in the main text with the data presented in these tables.

Comment # 3: The discussion was well considered and raised the interesting prospect that there may be non-immune effects of helminth infection which are mediating the protective effect that is seen in disease.

However, I was surprised that the authors did not mention or refer to the recent systematic review which assessed the association of helminth infection with the metabolic syndrome and diabetes (doi: 10.3389/fendo.2021.728396.) – this should be considered in their discussion in comparison the findings presented here.

**Response to Comment #3:** We thank the reviewer for bringing our attention to this recent systematic review, which assessed the association of helminth infection with metabolic syndrome and diabetes. We have now included this review (as well as a second review) in our introduction and discussion, starting at lines 117 and 920, respectively.

**Reviewer # 2’s Comments:**

The study by Pham et al addressed a pertinent issue in their comprehensive systematic review which aimed to assess the reported effects of helminth infections and antihelminthic treatment on the development and/or severity of cardiometabolic diseases and risk factors. After analyzing 83 animal and human studies, the authors concluded that helminth infection may offer protection against dyslipidemia, metabolic syndrome, diabetes, and atherosclerotic cardiovascular disease.  Overall, the manuscript is well written and can be further enriched by addressing the following comments:

Comment #1: Method: Clarify the statement "search was updated on March 1, 2022" versus data bases included until March 02, 2022.

**Response to Comment #1:** We thank the reviewer for this attention to detail. We reran the search on March 2 and have now corrected this in the manuscript in the abstract (lines 36-37) and methods (lines 139-140), as well as in Appendix 1.

Comment #2: Introduction: a bit shallow; better to show existing inconsistency in the literature though several studies favor inverse association between helminth infections and metabolic syndrome.

**Response to Comment #2:** We agree with the reviewer and have now included in our introduction section two other systematic reviews as well as a few other studies that show inconsistency in the literature (lines 117-121)

Comment #3: General questions on the discussion: Different helminths modulate the immune system differently and hence their effect varies. Besides, their effect could differ during acute and chronic infections with the typical example of schistosomiasis where infection with S mansoni for example induces production of both pro-inflammatory and anti-inflammatory as well as regulatory cytokines during acute and chronic infections, respectively. Did authors notice any difference between those studies which demonstrated inverse association versus those which didn’t in terms of chronicity of helminthic infections? Have authors noticed any common feature or limitation among those studies which reported the reverse or found no difference between infection and cholesterol level for example?

**Response to Comment #3:** Thank you for these questions. Some of the animal studies (Góes 2012, Phiri 2007, Saule 2005, Laothong 2013) showed different effects of helminth infections on cardiometabolic measures or outcomes at different stages or time points of infection. There were no reliably identifiable trends with regards to the chronicity of infection and its impact on the outcome. With regards to lipids and the reviewer’s last question, animal studies seemed to more consistently report inverse associations between helminth infections and lipid levels regardless of sex. Human studies reporting on lipids seem to be more inconsistent, and older women were particularly underrepresented in some studies that showed no or inconsistent associations between helminth infections and various lipid levels. We have highlighted this in the manuscript (lines 446-452).

Comment #4: Authors suggested non-immunological mechanisms/other metabolic mechanisms could explain the protective role of helminth infections based on their observation of the lack of association with hsCRP levels. Although CRP is an acute phase reactant and marker of inflammation, will it be valid to exclude immunological mechanisms without looking into the other immunological markers? It is very true that one of the key roles of the liver is lipid and glucose metabolism; however, this same organ is the source of many of the cytokines including those that affect the metabolic functions of the liver. Given the cross-sectional nature of the studies, establishing causal relationship might be difficult and the metabolic mechanisms suggested by authors (which are not explained) have to be considered without excluding immunological causes.

**Response to Comment #4:** We appreciate and agree with this thoughtful comment from reviewer #2. We had mentioned in the Discussion section (starting on line 938) that CRP is a nonspecific inflammatory marker and cannot represent the full complexity of immune responses. We have now revised and further emphasized this point by adding, “We note that CRP is a nonspecific inflammatory marker [(107)](https://sciwheel.com/work/citation?ids=1382709&pre=&suf=&sa=0&dbf=0) and we cannot conclude that lack of differences in CRP indicates lack of contribution of immune findings to our observations. However given the key role of the liver in lipid and glucose metabolism and the predilection of many helminths to affect this organ, we posit that altered intra-hepatic or other metabolic functioning may also contribute to the associations we have observed. One other important caveat is that we cannot exclude other immunologic mechanisms, including the liver’s role in host immune responses [(108)](https://sciwheel.com/work/citation?ids=2923054&pre=&suf=&sa=0&dbf=0), in analyzing the etiologies that may explain the inverse associations that we have reported” (lines 938-945).

Comment #5: Finally, given the fact that WHO recommends periodic deworming to all at-risk people living in endemic areas, what will be the implications of the findings?  Considering the protective role of helminthic infections can we recommend differently or where shall we strike the balance between keeping our old day worms and hence benefit from their co-existence versus their elimination (keeping in mind their bad side)?

**Response to Comment #5:** We appreciate this interesting and challenging question, given that there are probably not yet enough clinical data or experience to help strike a balance between harvesting benefits from helminths while avoiding the harmful effects from a chronic infection. For now, we can suggest that increased preventive care may be needed for individuals already at moderate or high risk for cardiometabolic disease who receive mass drug administration for helminth infections (lines 1043-1048). Further we can suggest the important need for, cross-disciplinary research to determine cardiometabolic effects of anthelmintic treatment, given that these emerging data would have important implications for both individual and population health and could help to address the challenges of managing non-communicable diseases in regions of the world that are endemic for helminthiasis. We have now strengthened this recommendation on lines 1048-1052.

Comment #6: Minor: please check reference listing format of the journal, some contain month. In addition, there are paragraphs/sentences throughout the document where reviewed studies’ findings are mentioned but with no references cited.
Other than these issues, this is an excellent comprehensive systematic review which revisits "the Hygiene hypothesis" with a different perspective.
Thank you

**Response to Comment #6:** We thank reviewer #2 again for taking the time to read through our manuscript and provide such thoughtful comments. We have checked our reference list and entire document using a reference manager to ensure that references are cited appropriately. We have now also added references, as applicable, throughout the results section where reviewed studies’ findings are mentioned.

**Reviewer # 3’s Comments:**

In this manuscript, Pham and colleagues present findings of a systematic review investigating the effects of helminth infection and anthelminthic treatment on cardiometabolic diseases and risk factors. They analysed eighty-three human and animal studies. These studies investigated the impact on lipids, metabolic syndrome, diabetes, blood pressure, atherosclerotic cardiovascular disease, high-sensitivity C-reactive protein, and non-atherosclerotic cardiovascular disease. The review shows that helminth infection may protect against cardiometabolic diseases and anthelminthic treatment may unravel this protection. These are very important findings for the discipline investigating the link between helminths and non-communicable diseases. The review is comprehensive covering both animal and human studies. Here are my concerns.

Comment #1: The authors need to revise the claim about the novelty of this review. There have been prior systematic reviews investigating the impact of helminths on metabolic outcomes such as Tracey et al *Do worms protect against the metabolic syndrome? A systematic review and meta-analysis* (DOI: 10.1016/j.diabres.2016.08.014) and Rennie et al *The Impact of Helminth Infection on the Incidence of Metabolic Syndrome: A Systematic Review and Meta-Analysis* (DOI: 10.3389/fendo.2021.728396). These should at least be cited or acknowledged either in the introduction or discussion of the review.

**Response to Comment #1:** We thank reviewer #3 for pointing out these other systematic reviews. We have now added these 2 other reviews to our introduction and discussion section, noting that our review built on these by including many more studies (both human and animal; 84 studies in ours vs. 4 and 14, respectively, in the two previous reviews), reporting on other cardiometabolic measures in addition to diabetes and metabolic syndrome, and additionally investigating the effects of anthelmintic treatment on these cardiometabolic outcomes. We also note that similar to these 2 previous reviews, we also found an inverse association between helminth infections and metabolic syndrome (starting at lines 117 and 920 in the introduction and discussion sections, respectively).

Comment #2: The authors have done a commendable job of searching the literature. It in ours would be nice to state, in the manuscript, the particular time frame covered by the review. Were the databases searched from inception? This is mentioned in the PROSPERO registration but not in the manuscript.

**Response to Comment #2:** Thank you for this comment. The databases were searched from inception (now added to the methods section) and the timeframe covered by the review has been clarified (lines 139-142).

Comment #3: For the PRISMA flow diagram, the authors should be more explicit about the exclusion of articles based on the eligibility criteria. Can the authors provide a breakdown of the particular number of articles exclude for each exclusion criterion, especially for the full-text articles?

**Response to Comment #3:** This is an important comment and we have added the breakdown of the number of articles excluded for each exclusion criterion for full-text articles—this is now part of the PRISMA flow diagram (pages 11-12).

Comment #4: Also limiting the search to the major helminths has resulted in the exclusion of important work done with other helminth species. For example the impact of *Nippostrongylus brasilie*nsis on serum cholesterol (Farid et al 2010 DOI 10.1111/j.1365-2362.2010.02352.x.0, ES-62, a product from the filarial nematode *Acanthocheilonema viteae* and atherosclerosis (Aprahamian et al., 2015 doi: 10.1016/j.ijpara.2014.12.006.) and the effect of ivermectin treatment of the nematode *Metastrongylus* spp. on triglyceride levels (Lopez-Olvera et al., 2006 doi: 10.1007/s00436-005-0099-2.) This should be acknowledged. Furthermore, the review seems to have missed important early contributions on the impact of Schistosoma mansoni on lipids such as de Oliveira et al. 1977 (doi: 10.1016/0014-4894(77)90029-7) and Baumheuer et al. 1994 (Trop Med Parasitol. 1994 Mar;45(1):5-8). The authors should address that.

**Response to Comment #4**: We thank reviewer #3 for making this important observation. Because helminths are such a diverse group and our search yielded a very large number of articles, we made the decision to limit our search to those that are most prevalent in humans. While we did come across important work done involving *Nippostrongylus brasiliensis*, *Acanthocheilonema viteae*, and *Metastrongylus* spp, these helminths are not known to be prevalent or pathogenic in humans, to our knowledge, and typically only infect rodents or pigs. Therefore, we did not think it was feasible to include all helminths. We have now mentioned this in our limitations section (lines 1032-1035).

We initially excluded these two other important contributions but now on second review, we agree and have added de Oliveira et al. 1977 (doi: 10.1016/0014-4894(77)90029-7) to our review. However, the second article did not meet criteria because it was less clearly focused on clinical lipid measurements, such as total cholesterol, LDL, HDL, or triglycerides. We have now also adjusted Figure 1 to reflect these changes.

Comment #5: Please report the animal and human studies separately, preferably with sub-headings, to clearly bring out the evidence. It will make it easier for the reader to decipher that the protective link between helminths and cardiometabolic outcomes has been demonstrated more clearly in animal studies than in human studies.

**Response to Comment #5:** We very much appreciate this comment from reviewer #3. In line with what was also suggested by reviewer #1, we have now included subheadings in our manuscript to delineate between animal and human studies in sections pertaining to studies examining a particular outcome before and after anthelmintic therapy, where appropriate and applicable.

As also discussed in response to Reviewer 1, most of the other sections (e.g., atherosclerotic cardiovascular disease, blood pressure and cardiovascular hemodynamics, hsCRP, and non-atherosclerotic cardiovascular disease) were already separated by human vs. animal study, and we believe that further adding subheadings may affect the flow of the manuscript. Similarly, for the cross-sectional studies in the other 3 sections (e.g., lipids, metabolic syndrome, and diabetes), we have included the number of human vs. animal studies in parentheses where it was important to delineate between the studies. For all cross-sectional and prospective or other studies, we have also organized Tables 2-8 to newly separate human and animal studies, so that readers can quickly glance at these tables as they read through the manuscript. We have additionally added the reference numbers for every study in Tables 2-8 to further assist the reader with matching the cited studies in the main text with the data presented in these tables.

Comment #6: As part of the reporting guidelines for systematic reviews, the authors should include a PRISMA checklist as supplementary material. Overall, this systematic review has important findings and is well-written. I would recommend the manuscript for publication after the comments above are addressed.

**Response to Comment #6:** This is an excellent point and we have now added a PRISMA checklist as supplementary material. We thank reviewer #3 again for taking the time to thoroughly review our work.

In reviewing and revising our paper, we also realized that our previous hypothesis about arterial stiffness and atherosclerosis, as they may potentially relate to helminths, was not entirely accurate. We have now revised this (lines 966-972).

We thank *PLOS Neglected Tropical Diseases* and its editors and peer reviewers again for considering our work.

Sincerely,

Khanh Pham, MD, MS, on behalf of all co-authors

Instructor in Medicine

Division of Infectious Diseases

Weill Cornell Medicine

Email: khp9007@med.cornell.edu or knpham11@gmail.com
